# Thieno[2,3-*b*]pyridines as a Novel Strategy Against Cervical Cancer: Mechanistic Insights and Therapeutic Potential

**DOI:** 10.3390/ijms26062651

**Published:** 2025-03-14

**Authors:** Monika Čikeš Botić, Sandra Marijan, Mila Radan, Ivana Novak, Mateo Glumac, Lisa I. Pilkington, Zdravko Odak, David Barker, Jóhannes Reynisson, Vedrana Čikeš Čulić

**Affiliations:** 1 Department of Gynecology and Obstetrics, University Hospital of Split, 21000 Split, Croatia; zodak@kbsplit.hr; 2 Department of Medical Chemistry and Biochemistry, School of Medicine, University of Split, 21000 Split, Croatia; sandra.marijan@mefst.hr (S.M.); vedrana.cikes.culic@mefst.hr (V.Č.Č.); 3 Department of Biochemistry, Faculty of Chemistry and Technology, University of Split, 21000 Split, Croatia; mradan@ktf-split.hr; 4 Department of Immunology and Medical Genetics, School of Medicine, University of Split, 21000 Split, Croatia; ivana.novak.nakir@mefst.hr (I.N.); mglumac@mefst.hr (M.G.); 5 School of Chemical Sciences, The University of Auckland, Auckland 1010, New Zealand; lisa.pilkington@auckland.ac.nz (L.I.P.); d.barker@auckland.ac.nz (D.B.); 6 Te Pūnaha Matatini, Auckland 1042, New Zealand; 7 The MacDiarmid Institute for Advanced Materials and Nanotechnology, Wellington 6012, New Zealand; 8 School of Allied Health Professions and Pharmacy, Keele University, Staffordshire ST5 5BG, UK; j.reynisson@keele.ac.uk

**Keywords:** cervical cancer, cancer stem cells (CSCs), cytotoxicity, thieno[2,3-*b*]pyridines, apoptosis, glycosphingolipids (GSLs), metabolomics

## Abstract

Cervical cancer is the fourth leading cause of cancer mortality in women worldwide, with limited therapeutic options for advanced or recurrent cases. In this study, the effects of a recent thieno[2,3-*b*]pyridine derivative, (E)-3-amino-5-(3-bromophenyl)acryloyl)-*N*-(3-chloro-2-methylphenyl)-6-methylthieno[2,3-*b*]pyridine-2-carboxamide (compound **1**), on two cervical cancer cell lines, HeLa and SiHa, are investigated. Cytotoxicity was assessed by MTT assay, apoptosis rates were measured by flow cytometry, and metabolic profiling was performed by GC-MS. The study also examined the expression of eight glycosphingolipids (GSLs) in cancer stem cells (CSCs) and non-CSCs to assess glycophenotypic changes. Compound **1** showed significant cytotoxicity in both cell lines, with apoptosis identified as the primary mechanism of cell death. A significant reduction in the CSC population was observed, particularly in the SiHa cell line. Compound **1** treatment altered GSL expression and decreased GM2 levels in both CSCs and non-CSCs in the SiHa cell line and Gg3Cer levels in the HeLa cell line. Metabolic profiling identified 23 and 21 metabolites in the HeLa and SiHa cell lines, respectively, with significant differences in metabolite expression after treatment. These results underscore the potential of compound **1** as a promising therapeutic candidate for cervical cancer and warrant further investigation in preclinical and clinical settings.

## 1. Introduction

Cervical cancer is the fourth most frequently diagnosed cancer (6.8%) and the fourth leading cause of cancer-related deaths (8.1%) among women in 2022 based on the GLOBOCAN estimates of cancer incidence and mortality [1]. The primary etiologic agent of cervical cancer is human papillomavirus (HPV) infection [2]. Incidence and mortality are higher in developing countries due to their lower human development index, socioeconomic status, and healthcare expenditure [1,3]. Treatment for cervical cancer varies based on the stage of the disease. For early-stage cervical cancer, an appropriate treatment is conization, hysterectomy, or chemoradiotherapy, depending on the International Federation of Gynecology and Obstetrics (FIGO) classification. For locally advanced cervical cancer, the preferred treatment is combined chemoradiotherapy and brachytherapy. Likewise, chemoradiotherapy combined with immunotherapy is a preferred treatment option for recurrent or metastatic cervical cancer [4,5]. The inspiration for this research is women who experienced delayed detection of the disease. In that population, efficient treatment modalities are very restricted.

Cancer stem cells’ (CSCs) resistance is typically associated with chemoresistance in general [6]. CSCs represent a minor subpopulation of cells possessing pluripotent or multipotent capabilities characterized by their capacity for proliferation, differentiation, and remarkable phenotypic plasticity. For this reason, CSCs are the primary therapeutic target in cancer treatment. The surface markers of cervical CSCs are characterized by the following biomarkers: CD133, CD34, CD44, CD49f, CD26, and CD90 [7]. Liu et al. demonstrated that a subpopulation of human cervical cancer cells with elevated ALDH activity exhibit increased self-renewal capacity, differentiation potential, and enhanced tumorigenicity, suggesting that high ALDH activity could serve as a marker for CSCs in cervical cancer [8]. High-risk HPV targets cervical epithelial stem cells exhibiting the surface markers listed above. CD49f (α-6 integrin) is one of the essential co-receptors found to be upregulated in HPV-infected cervosphere cells and CD49f-positive cells have enhanced tumorigenic capabilities [9,10]. CD49f-positive cells also exhibit increased proliferative potential and high CD49f expression is linked to both lymph nodal and distant metastatic spread [11]. In colorectal cancer, CD166^+^/CD44^+^ cells demonstrated greater clonogenicity and faster tumor development compared to CD166^−^CD44^−^ cells, with this observation being dependent on the cell line [12]. Another study integrated CD133 with CD44 to identify lung CSCs in A549 cells, finding that CD133^+^/CD44^+^ cells exhibited sustained proliferative capacity and differentiation potential [13]. Apoptosis or programmed cell death is an energy-dependent biochemical mechanism that expresses distinct morphological changes. Cell turnover, development, and immune system function are essential components of normal cell action, etc. Aberrant apoptosis indicates diseases, e.g., neurodegenerative diseases, autoimmune disorders, and numerous types of cancer. The ability to modulate cell death has a huge therapeutic potential [14].

Metabolomics has emerged as a promising tool for therapeutic and diagnostic oncology applications. The metabolome, comprising a diverse array of small chemical molecules known as metabolites, plays a pivotal role in all physiological mechanisms and disease states. In the context of cervical cancer, metabolomic profiling has revealed significant alterations in metabolite levels between healthy individuals and patients, highlighting the potential of these molecules as candidate biomarkers for diagnosis. A comprehensive study identified statistically significant differences in several metabolites, including bilirubin, LysoPC(17:0), *n*-oleoyl threonine, 12-hydroxydodecanoic acid, and tetracosahexaenoic acid, in cervical cancer patients compared to healthy controls [15]. Further research by Nuer-Allornuvor et al. proposed a two-biomarker panel consisting of 2-methyl-1-propylamine (isobutylamine) and estrone as a potentially effective method for early diagnosis of cervical cancer (CC), particularly in the early stages of cervical intraepithelial neoplasia (CIN I and II) [16]. Their findings demonstrated elevated levels of 2-methyl-1-propylamine across numerous pathological conditions (CC, CIN I, CIN II, CIN III) relative to healthy subjects, while estrone levels were diminished in all these conditions compared to healthy volunteers. It is important to note that chemotherapy can induce significant metabolic alterations, further underscoring the complexity of metabolomic profiles in cancer patients and the potential for metabolomics in monitoring treatment responses and disease progression.

Glycosphingolipids (GSLs) are integral components of cell plasma membranes that participate in cellular signaling, proliferation, apoptosis, adhesion, recognition, and cancer metastasis. There can be a variation in GSL expression between CSCs and non-CSC(s) [17]. Notably, there are certain types of GSLs that are more abundantly expressed in tumors than in normal tissues [18]. For this reason, GSLs are known also as tumor-associated antigens. It has been shown that most cancer cells show amended GSL patterns, abnormal GSL signaling, and biosynthesis, which combined play a major role in tumor progression. Tumor progression is dependent on angiogenesis, a process that involves the formation of new blood vessels that grow into the tumor in response to factors released by the tumor itself. For instance, gangliosides such as GM2, GM3, and GD3 influence the epidermal growth factor receptor (EGFR), while GM3 and GD3 affect the vascular endothelial growth factor receptor (VEGFR) [19]. Tumor-associated GSL antigens have been used in the development of antitumor vaccines [18]. Undoubtedly, GSLs play a crucial part in tumor development and are a promising aim for cancer treatment.

**Figure 1:**
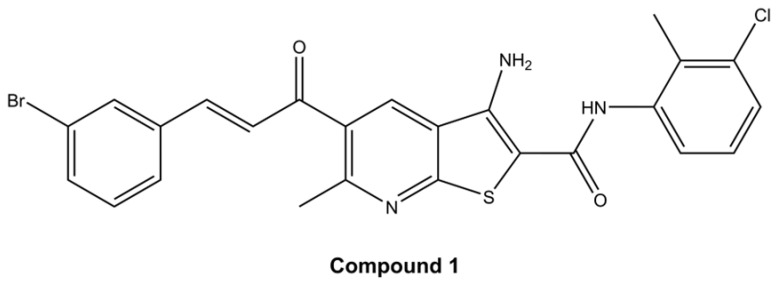
Structure of (*E*)-3-amino-5-(3-3-bromophenyl)acryloyl)-*N*-(3-chloro-2-methylphenyl)-6-methylthieno[2,3-*b*]pyridine-2-carboxamide (compound **1**).

The family of thieno[2,3-*b*]pyridines are inhibitors of phospholipase C isoforms that have been shown to be effective anticancer compounds against many cancer cell lines [20]. Indeed, our previous research confirms the anticancer activity of this compound class. Namely, it was shown that in ovarian cancer thieno[2,3-*b*]pyridines induce apoptosis, reduce the number of CSCs, alter GSL and metabolite expression [21]. Furthermore, in triple-negative breast cancer cells, they induce apoptosis, reduce CSC numbers, and modify GSL expression [22]. The aim of this research was to explore the potential chemotherapeutic effect of new thieno[2,3-*b*]pyridine on cervical CSCs by examining cytotoxicity, apoptosis, metabolomics, GSL expression, and their ability to reduce pluripotency of cervical cancer stem cells by shifting the glycophenotype. A compound (*E*)-3-amino-5-(3-3-bromophenyl)acryloyl)-*N*-(3-chloro-2-methylphenyl)-6-methylthieno[2,3-*b*]pyridine-2-carboxamide (compound **1**, Figure 1) was selected for this research due to its enhanced potency against HeLa and SiHa cell lines and its mode of action has been investigated [23]. Eight GSLs (Gg_3_Cer, Gb_4_Cer, nLc_4_Cer, GM3, GD3, GM2, GalNacGM1b, and IV^3^Neu5Ac-nLc_4_Cer) were examined, and their expression was compared between CSCs and non-CSCs after compound **1** treatment.

## 2. Results

### 2.1. Cell Viability

**Figure 2:**
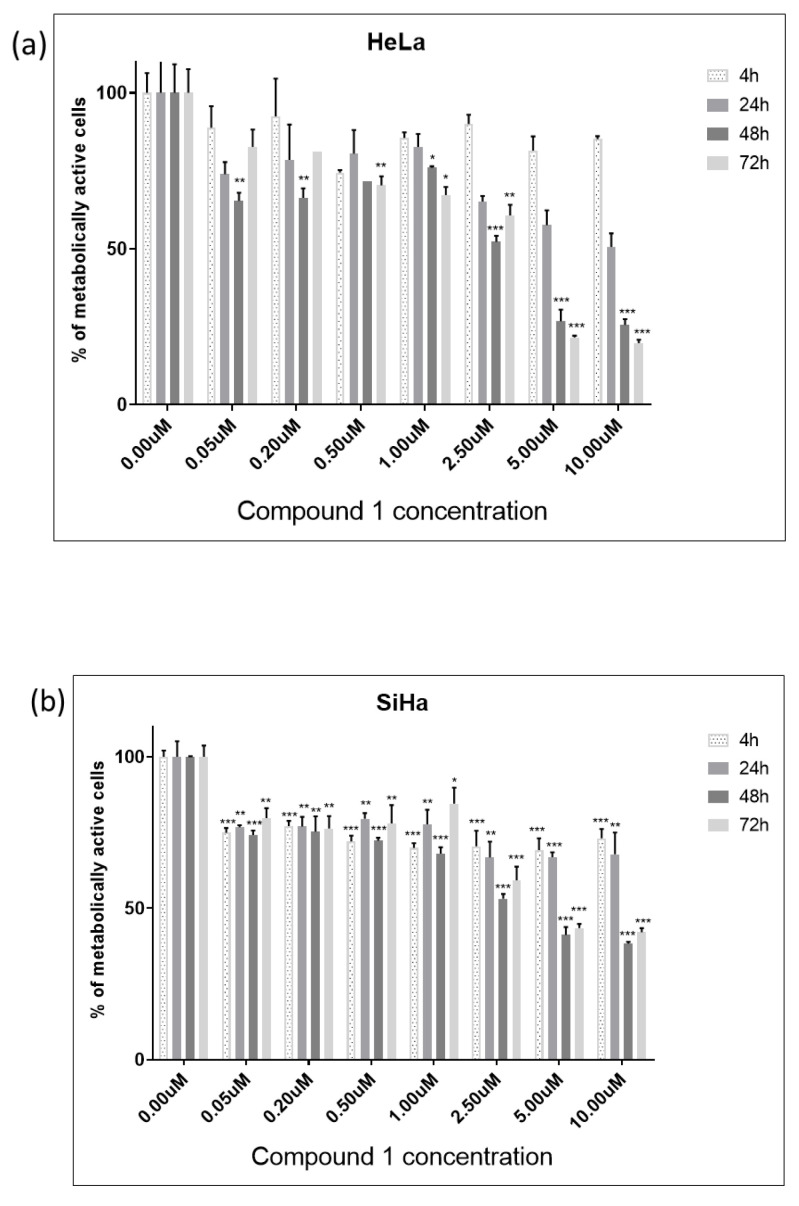
Cell viability of HeLa (**a**) and SiHa (**b**) cell line after treatment with compound **1**. Notes: Data are expressed as a mean from the experiment performed in triplicate ± SD. Columns, mean of metabolically active cells; bars, SD (standard deviation); *p*-values relate to a two-sample *t*-test comparing the treatment at the given concentration with when no treatment is applied (0 µM, control); * p-value < 0.05; ** *p*-value < 0.01; *** *p*-value < 0.001.

Cell viability was evaluated by employing the MTT assay which determines the cell metabolic potential. Cells from both cell lines, HeLa and SiHa, were treated with four different thieno[2,3-*b*]pyridines (see Figure 1 and Supplementary Files—Figures S1, S4 and S8 for structures). The cells were exposed to 7 different concentrations (0.05 µM, 0.2 µM, 0.5 µM, 1 µM, 2.5 µM, 5 µM, and 10 µM) of each cytotoxic agent for 4, 24, 48 and 72 h (see *p*-values in Supplementary Files—Tables S1, S3, S5, S7, S9, S11, S14 and S16). The half-maximal effective concentration (EC_50_) was calculated for all four time-points (data shown in Supplementary Files—Tables S2, S4, S6, S8, S10, S12, S15 and S17). Due to the highest efficiency, e.g., lowest EC_50_, compound **1** was chosen for further testing (see Supplementary Files—Figures S2, S3, S5, S6, S9 and S10 for cell viability of HeLa and SiHa cell lines after treatment with others compounds). After 48 h of treatment with compound **1**, EC_50_ was 2.14 µM and 2.77 µM for HeLa and SiHa cell lines, respectively. In comparison, clinically used cisplatin is reported to have EC_50_ 21.3 µM and 80 µM in HeLa and SiHa cell lines, respectively, when tested under similar conditions [24,25]. Also, for the HeLa cell line, EC_50_ was 32.82 µM after 4 h, 6.30 µM after 24 h, and 2.11 µM after 72 h. For the SiHa cell line, EC_50_ was 11.51 µM, 10.06 µM, and 4.12 µM after 4 h, 24 h, and 72 h, respectively (Figure 2).

In the HeLa cell line, compound **1** was shown to be cytotoxic in 0.05 µM concentration after only 4 h of treatment (Figure 2a). After 48 h of treatment, the concentration of 2.5 µM was cytotoxic to more than 50% of cells. The maximum cytotoxic effect was observed after 72 h of treatment when exposed to a concentration of 10 µM of compound **1**.

Furthermore, in the SiHa cell line, more than 50% of cells were metabolically inactive after 48 h of treatment with a concentration of 2.5 µM of compound **1** (Figure 2b). However, compound **1** demonstrated a slightly diminished effectiveness in this cell line, but cytotoxicity demonstrated a proportional increase with concentration. The maximum cytotoxic effect in the SiHa cell line was observed after 48 h of treatment when exposed to a concentration of 10 µM of compound **1**.

**Figure 3:**
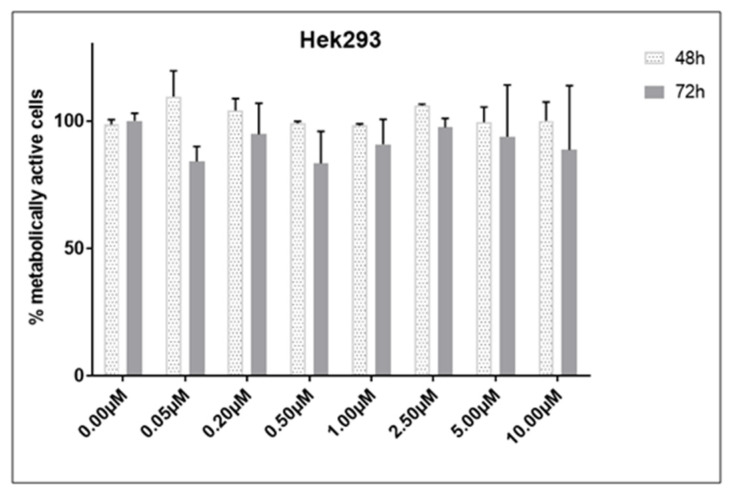
Cell viability of HEK-293 cell line after treatment with compound **1**. Notes: Data are expressed as a mean from the experiment performed in triplicate ± SD. Columns, mean of metabolically active cells; bars, SD (standard deviation).

We have employed human embryonic kidney 293 cells (HEK-293) as a non-cancerous, normal cell line to test the specificity of compound **1** towards cancer cells. The cytotoxicity of compound **1** was tested on HEK-293 cells, revealing no significant effect on normal cells, as demonstrated in the graph (Figure 3). Over 90% of the cells remained viable across all concentrations and incubation durations.

### 2.2. Apoptosis Rate

**Figure 4:**
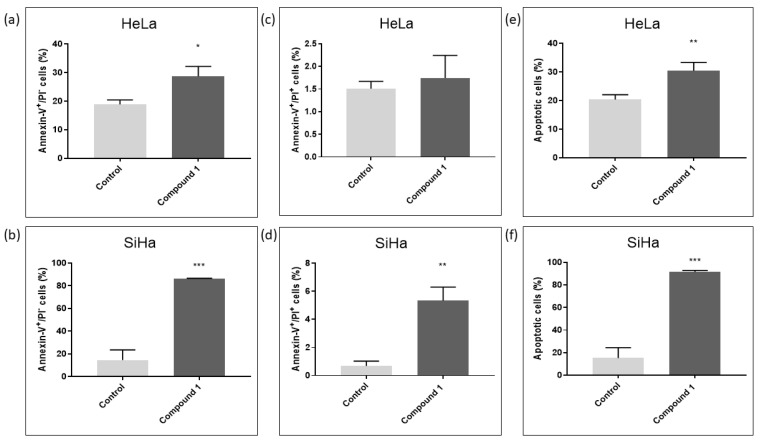
Apoptosis after treatment with compound **1**. Notes: Percentage of cells in early (**a**), late (**c**), and total (**e**) apoptosis without and with compound **1** treatment for 48 h in the HeLa and early (**b**), late (**d**), and total (**f**) apoptosis in the SiHa cell lines. Data represented are expressed as a mean from the experiment performed in triplicate ± SD. Columns, mean of cells; bars, SD; *p*-values relate to a two-sample *t*-test comparing the treatment at the given concentration with when no treatment is applied (0 µM, control); * p-value < 0.05; ** *p*-value < 0.01; *** *p*-value < 0.001.

For the SiHa cell line, a concentration of 2.5 µM was utilized, while for the HeLa cell line, a concentration of 2.14 µM (EC_50_ concentration for 48 h of treatment) was employed to assess the effect of compound **1** on the percentage of early (Annexin-V^+^PI^−^ subpopulation), late (Annexin-V^+^PI^+^ subpopulation) and total apoptotic cells (Figure 4). HeLa cells were more resistant to compound **1** treatment compared to SiHa cells. The results for Hela cells show a statistically significant increase in both early and total apoptosis was noted after the treatment with compound **1**, 28.70 ± 3.45% (*p*-value < 0.05) and 30.44 ± 2.95% (*p*-value < 0.01), respectively, while there was no statistically significant difference between treated and untreated cells in late apoptosis.

In contrast, in the SiHa cell line, there was a statistically significant increase in early, late, and total apoptosis among treated cells compared to untreated cells. After treatment with compound **1,** there were 86.17 ± 0.49% early apoptotic cells (*p*-value < 0.001) compared to untreated 14.62 ± 8.88%, 5.33 ± 0.96% late apoptotic cells (*p*-value < 0.01) compared to untreated 0.68 ± 0.34% and 91.50 ± 1.32% total apoptotic cells (*p*-value < 0.001) compared to untreated 15.30 ± 9.11%.

**Figure 5:**
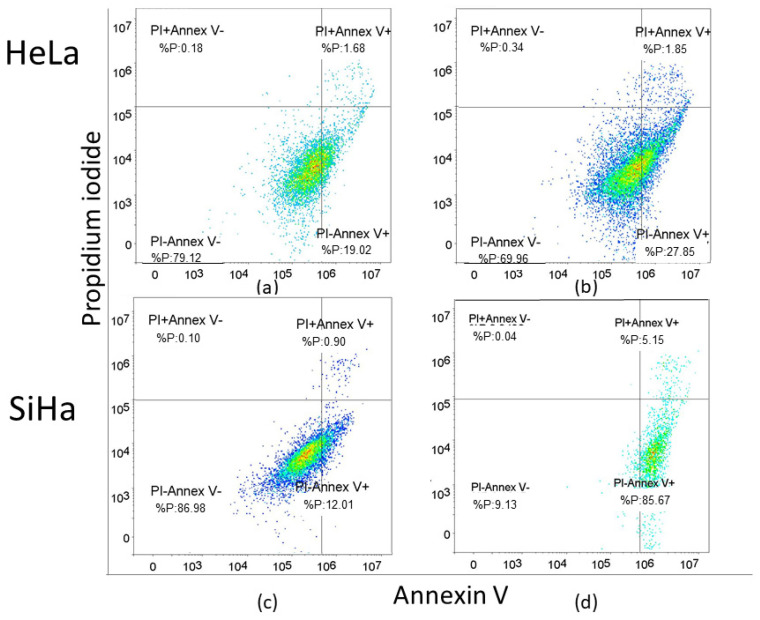
Apoptosis without and with the compound **1** treatment. Notes: Dot plots of apoptotic cells without and with compound **1** treatment for 48 h in the HeLa (**a**,**b**) and the SiHa cell line (**c**,**d**). The x-axis represents Annexin-V and the y-axis represents propidium iodide (PI).

Representative dot blot graphs demonstrate that treated cells exhibit a significant rise in early apoptosis (Annexin V^+^/PI^−^) compared to untreated cells in both HeLa and SiHa cell lines (Figure 5).

### 2.3. Cancer Stem Cells

**Figure 6:**
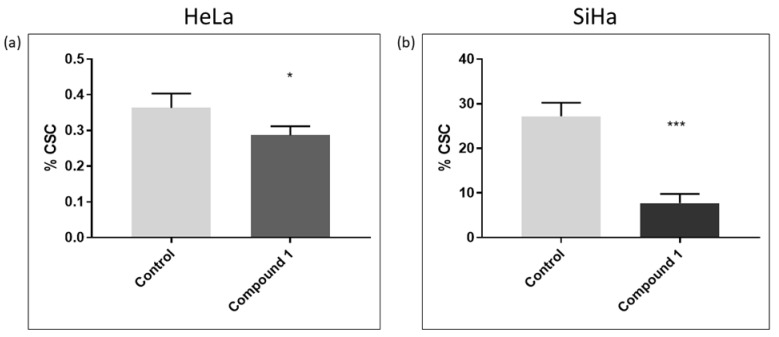
Cancer stem cells after 48 h treatment with compound **1**. HeLa cell line (**a**) and SiHa cell line (**b**). Notes: Data represented are expressed as a mean from an experiment performed in triplicate  ±  SD. Columns, mean of cells; bars, SD; *p*-values relate to a two-sample *t*-test comparing the treatment at the given concentration with when no treatment is applied (0 µM, control); * p-value < 0.05; *** *p*-value < 0.001.

In this study, the CD49f was used as a marker for cancer stem cells (CSCs) [9]. The CD49f^+^ subpopulation in both cell lines was analyzed to explore the potential impact of compound **1** on reducing the number of CSCs. There was a statistically significant reduction in the percentage of treated CSCs in the HeLa cell line (*p*-value < 0.05) compared to the control, although this reduction (0.36 ± 0.04% and 0.29 ± 0.02%, respectively) was less pronounced than in the SiHa cell line (Figure 6a). There was a statistically significant decrease in the percentage of treated CSCs (*p*-value < 0.001) compared to untreated ones (27.17 ± 3.05% and 7.75 ± 2.03%, respectively) (Figure 6b).

ALDH was used as another marker for cervical cancer stem cells [8]. It was used to verify whether these cells were indeed CSCs using ALDH, and nearly identical results were obtained (Figure S7—Supplementary File). There was a statistically significant reduction in the percentage of treated CSCs in the HeLa cell line (*p*-value < 0.001) compared to the control (0.08 ± 0.01% and 0.04 ± 0.00%, respectively) (Figure S7a). In the SiHa cell line, there was a statistically significant decrease in the percentage of treated CSCs (*p*-value < 0.001) compared to untreated ones (10.03 ± 0.92% and 2.32 ± 1.17%, respectively) (Figure S7b).

### 2.4. Expression of Glycosphingolipids

**Figure 7:**
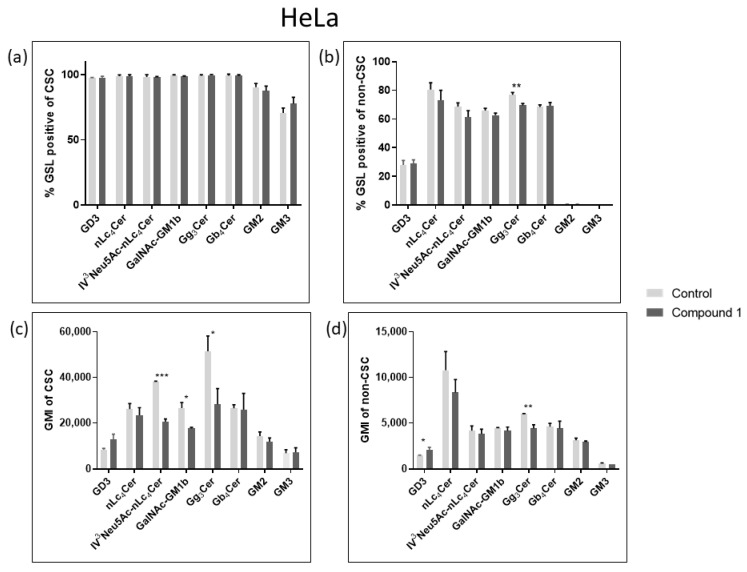
Percentage and geometric mean intensity of GSL-positive cells after 48 h treatment with compound **1** in the HeLa cell line. Percentage of GSL-positive CSCs (**a**) and non-CSCs (**c**). Geometric mean fluorescence intensity of CSCs (**b**) and non-CSCs (**d**). Notes: Data are expressed as a mean from the experiment performed in triplicate ± SD. Columns, mean of viable cells; bars, SD; *p*-values relate to a two-sample *t*-test comparing the treatment at the given concentration with when no treatment is applied (0 µM, control); * p-value < 0.05; ** *p*-value < 0.01; *** *p*-value < 0.001. CSC, CD49f^+^; non-CSC, CD49f^−^; GSL, glycosphingolipid; CSCs, cancer stem cells; GMI, geometric mean intensity; SD, standard deviation. The designation of the gangliosides adheres to the IUPAC-IUB recommendations [26] and the nomenclature established by Svennerholm [27]: Neu5Ac, N-acetylneuraminic acid; nLc4Cer, neolactotetraosylceramide; IV^3^Neu5Ac-nLc_4_Cer; globotetraosylceramide or Gb_4_Cer; gangliotriaosylceramide or Gg_3_Cer; GalNAc-GM1b; GM2.

**Figure 8:**
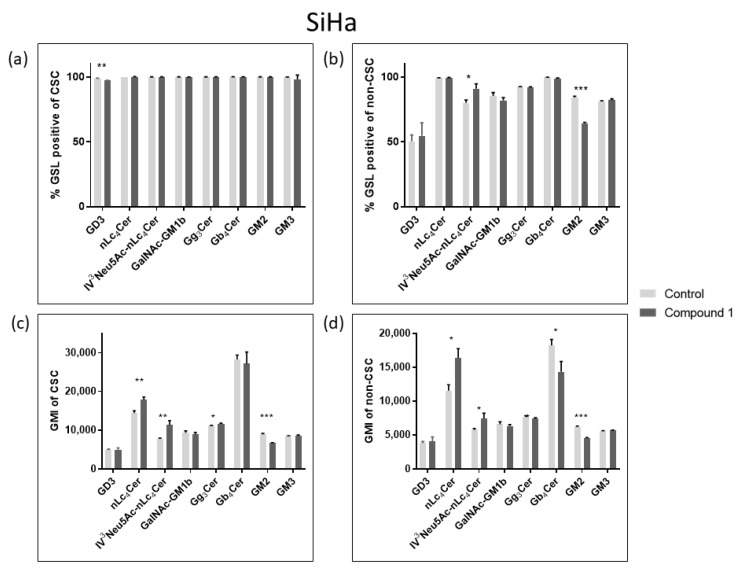
Percentage and geometric mean intensity of GSL-positive cells after 48 h treatment with compound **1** in the SiHa cell line. Percentage of GSL-positive CSCs (**a**) and non-CSCs (**c**). Geometric mean fluorescence intensity of CSCs (**b**) and non-CSCs (**d**). Notes: Data are expressed as a mean from the experiment performed in triplicate ± SD. Columns, mean of viable cells; bars, SD; *p*-values relate to a two-sample *t*-test comparing the treatment at the given concentration with when no treatment is applied (0 µM, control); * p-value < 0.05; ** *p*-value < 0.01; *** *p*-value < 0.001; CSC, CD49f^+^; non-CSC, CD49f^−^; GSL, glycosphingolipid; CSCs, cancer stem cells; GMI, geometric mean intensity; SD, standard deviation.

Considering their role in tumor signaling and progression, three neutral GSLs (Gg_3_Cer, Gb_4_Cer, and nLc_4_Cer) and five gangliosides (GM3, GD3, GM2, GalNAcGM1b, and IV^3^Neu5Ac-nLc_4_Cer) were investigated and their expression was compared between CSCs and non-CSCs after 48 h treatment with compound **1**. The goal was to determine whether the treatment with compound **1** affects GSL membrane compositions. The percentage and the geometric mean intensity (GMI), expression of each GSL per one cell, which is of greater importance, were analyzed (Figure 7 and Figure 8).

In the HeLa cell line, only the percentage of Gg_3_Cer-positive cells were significantly decreased in non-CSCs, from 76.94% to 69.58% (Figure 7c). Likewise, compound **1** decreased GMI of Gg_3_Cer-positive cells in both CSCs and non-CSCs, from 51,472 to 28,272 and from 6030 to 4408, respectively (Figure 7b,d).

In the SiHa cell line, treatment with compound **1** resulted only in a significant percentage decrease in GD3-positive CSCs, from 98.75% to 97.04% (Figure 8a). On the other hand, there was a statistically significant percentage increase in IV^3^Neu5Ac-nLc_4_Cer-positive cells, from 79.93% to 90.9%, and a decrease in the percentage of GM2-positive non-CSCs from 84.23% to 64.2% (Figure 8c). After exposure to Compound **1**, there was an increased GMI of nLc_4_Cer-positive CSCs, from 14,426 to 17,852, and nLc_4_Cer-positive non-CSCs from 7746 to 11,320. The same result was observed for IV^3^Neu5Ac-nLc_4_Cer-positive CSCs, GMI increased from 11,540 to 16,373, and IV^3^Neu5Ac-nLc_4_Cer-positive non-CSCs from 5769 to 7430 after treatment with Compound **1**. However, the expression of GM2 was decreased in both CSCs and non-CSCs (from 8937 to 6753 and from 6199 to 4571, respectively) (Figure 8b,d).

### 2.5. Metabolites

Metabolic profiling was conducted utilizing GC-MS. In the HeLa cell line, 23 metabolites were detected, while in the SiHa cell line, 21 metabolites were identified (Table 1). Only metabolites listed in the Human Metabolome Database (HMDB 4.0) were chosen. The aim was to determine the impact of compound **1** on individual metabolites and to identify metabolites that demonstrated significant differences between treated and untreated cells. In each cell line, HeLa and SiHa, several metabolites were found to be in different amounts in treated and untreated cells, but only one metabolite was statistically different (*p*-value < 0.05) from the control group in each of the cell lines, glycerol and octadecanol, respectively.

**Figure 9:**
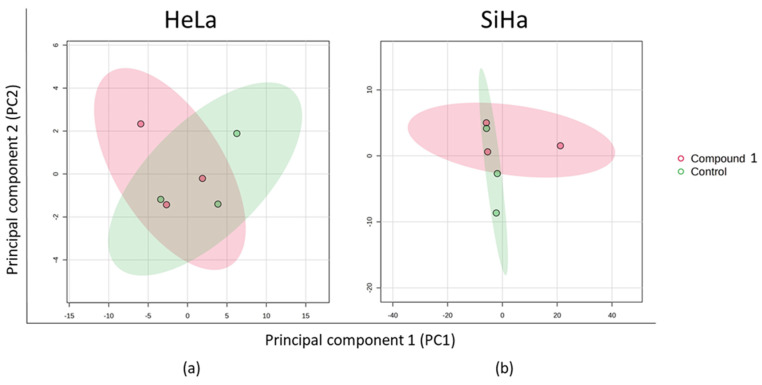
PCA of the metabolic profile after 48 h treatment with compound **1**. Notes: PCA of metabolic profile of HeLa (**a**) and SiHa (**b**) cell lines. PCA, principal component analysis.

The principal component analysis (PCA) plot transforms the correlations among cells into a 2D graph, where cells with similar metabolite profiles are shown to be clustered together. The axes in the plot are ordered by importance, with differences along the first principal component axis (PC1) considered more significant than those along the second principal component axis (PC2), with the higher value of data variability explained by PC1. In the HeLa cell line, PC1 accounted for 79.9% of the variance and PC2 for 10.4% (Figure 9a). For the SiHa cell line, PC1 explained 80.6% of the variance while PC2 explained 18.5% (Figure 9b). Both plots showed that treatment with compound **1** led to changes in metabolite expression.

**Figure 10:**
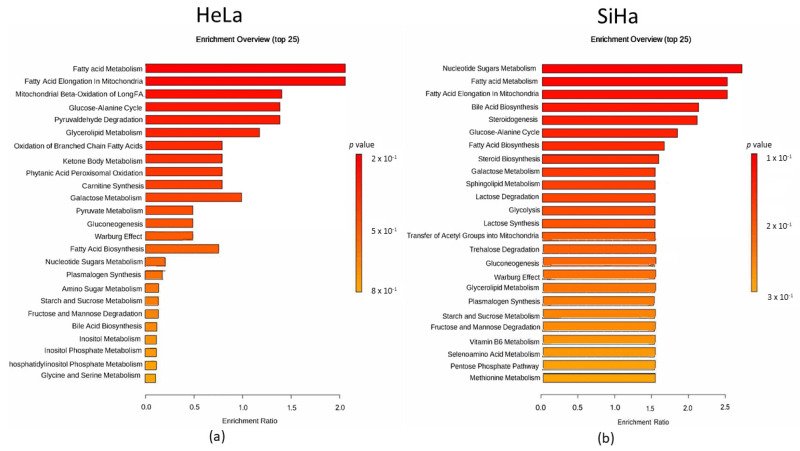
Metabolomic set enrichment analysis after the 48 h treatment with compound **1**. Note: Metabolomic enrichment analysis of HeLa (**a**) and SiHa (**b**) cell lines.

Quantitative enrichment analysis was utilized to detect patterns in metabolite concentrations and to aid in uncovering potential biological mechanisms. Results were considered statistically significant with *p*-value < 0.05, but no statistical difference was found for either of the cell lines (Figure 10). In both cell lines, fatty acid metabolism and fatty acid elongation in mitochondria were most prominent. Specifically, mitochondrial β-oxidation of long-chain saturated fatty acids, the glucose-alanine cycle, and pyruvaldehyde degradation were notable in the HeLa cell line. In the SiHa cell line, nucleotide sugar metabolism, bile acid biosynthesis, and steroidogenesis were most represented.

**Figure 11:**
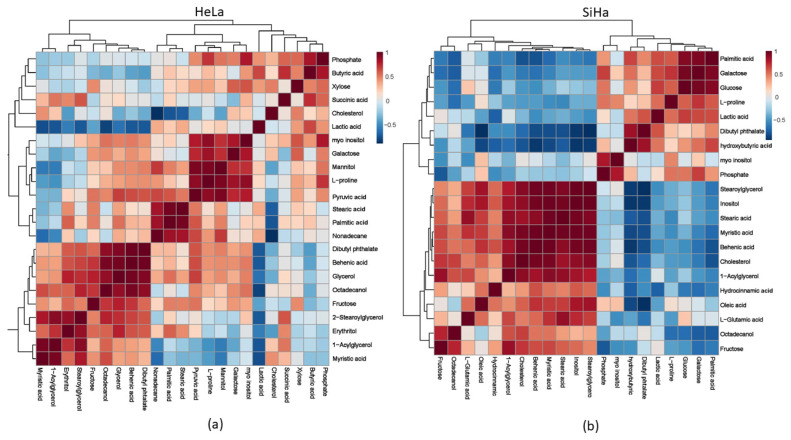
Metabolomic correlation matrix after the 48 h treatment with compound **1**. Notes: Metabolomic correlation matrix of HeLa (**a**) and SiHa (**b**) cell lines. The highest positive correlations are shown in dark red shades, while the highest negative correlations are in dark blue shades. “Clusters” of substances are connected outside of the matrix.

We demonstrated a more precise connection between metabolites in both cell lines after treatment with compound **1** by using a correlation matrix, depicted with a heat map (Figure 11).

### 2.6. Effect on Mitochondria

**Figure 12:**
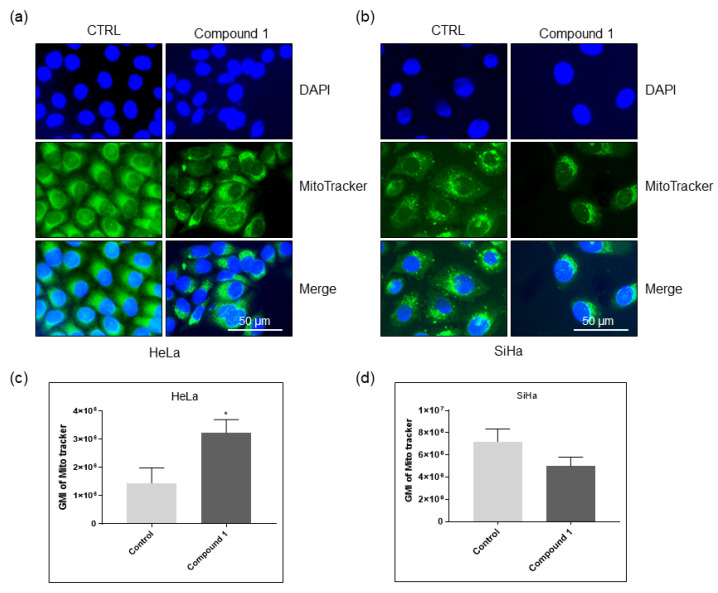
The effect of compound **1** on mitochondrial morphology and quantity. Notes: Immunofluorescence images of Hela (**a**) and SiHa (**b**) cells untreated and treated with compound **1** stained with Mitotracker. Geometric mean fluorescence intensity of Mitotracker in HeLa (**c**), and SiHa (**d**) cells, expressed as a mean from the experiment performed in triplicate ± SD. Notes: *p*-values relate to a two-sample *t*-test comparing the treatment at the given concentration with when no treatment is applied (0 µM, control); * p-value < 0.05; GMI, geometric mean intensity; SD, standard deviation.

Metabolite analysis suggests a strong impact on mitochondrial fatty acid metabolism and fatty acid elongation. To elucidate the impact of compound **1** on mitochondrial morphology and number, we performed mitotracker stainings. HeLa and SiHa cells were treated with compound **1** for 48 h and stained with mitotracker before observation. The staining revealed a strong impact of compound **1** on mitochondria in both cell lines (Figure 12). In both cell lines, mitochondria looked fragmented, suggesting mitochondrial fission. Furthermore, in both cell lines, mitochondrial distribution shifted, becoming more localized near the nucleus. We have also observed a striking difference in mitochondrial quantity between the two cell lines after treatment. In HeLa cells, the mitotracker signal appeared stronger in the treated cells compared to the control cells, whereas in the SiHa cells, the signal was diminished in treated cells. To quantify this effect, cells were analyzed by flow cytometry. As expected, the flow cytometry confirmed our observations. The mitoctracker signal is strongly increased in the HeLa cell line, while it shows a reduction in the SiHa cell line. This points to differential impact of compound **1** on mitochondria in these two cell lines. In Hela cells, compound **1** leads to accumulation of mitochondria, while in the SiHa cells, it leads to reduction in mitochondrial number, which could explain the differences in glycosphingolipid expression and metabolite levels.

## 3. Discussion

Cervical cancer represents a significant global health concern, especially in developing countries [1]. The Cervical Cancer Elimination Initiative has established national 90–70–90 targets for countries, aiming to put them on track towards the elimination of cervical cancer by 2030. It is essential that 90% of girls receive the complete HPV vaccination by the age of 15, 70% of women go through screening at ages 35 and 45, and 90% of women diagnosed with precancerous lesions or invasive cancer receive treatment. Despite the WHO’s global strategy to decrease incidence rates to below 4 per 100,000 women-years within this century, modeling studies suggest that achieving the elimination aim may not occur before the century’s end in these countries unless there is a substantial increase in preventive and curative interventions, such as HPV screening and vaccination [28,29].

Cervical cancer is the most prevalent gynecological malignity and a significant global health issue. It predominantly affects women in developing countries, but even in developed countries, cervical cancer continues to pose a risk. The prognosis for early-stage cervical cancer is optimistic, but for advanced-stage or recurrent disease, it is very poor [30]. SiHa cells, containing HPV-16, and HeLa cells, containing HPV-18, are epithelial cell lines derived from squamous cell carcinoma and adenocarcinoma, respectively. For cervical squamous cell carcinoma (SCC), especially in the advanced stage, platinum-based concurrent chemo-radiotherapy (CCRT) has been a standard therapy. In the recent study, after 48 h incubation with cisplatin, SiHa cells exhibited strong resistance, with an IC50 value of 17.37 µM [31]. Regarding CCRT in patients with cervical adenocarcinoma (ADC), no prospective studies have been conducted to date. However, retrospective studies have indicated poorer overall survival (OS) in ADC patients compared to those with SCC. ADC generally exhibits resistance to both radiation and chemotherapy [32]. Chemoradiotherapy combined with immunotherapy is a treatment for recurrent or metastatic cervical cancer. Yang et al. demonstrated that bevacizumab combined with neoadjuvant chemotherapy and platinum-based concurrent chemoradiotherapy notably enhanced total clinical response and overall survival in refractory cervical cancer [33]. However, most cervical cancer diagnoses occur in low- and middle-income countries, where many women cannot afford bevacizumab due to its high cost [34]. This research is motivated by women with cervical cancer who were not diagnosed early enough, facing severely limited treatment options.

To the best of our knowledge, this study represents the first investigation of the effects of thieno[2,3-*b*]pyridines on cervical cancer cell lines, HeLa and SiHa. We demonstrated the cytotoxic impacts of (*E*)-3-amino-5-(3-(3-bromophenyl)acryloyl)-*N*-(3-chloro-2-methylphenyl)-6-methylthieno[2,3-*b*]pyridine-2-carboxamide (compound **1**) on two cervical cancer cell lines listed above [23]. Recent studies have demonstrated that thienopyridines interact with a wide range of enzymes, including tyrosyl-DNA phosphodiesterase I (TDP1), A_2A_ receptor antagonists (A_2A_ AR), G-protein coupled receptors (GPCRs), P2Y12 receptors, copper trafficking proteins such as Atox, and tubulin. Consequently, the anti-proliferative activity of thienopyridines cannot be exclusively attributed to PI-PLC inhibition, although PI-PLC remains a validated and relevant target [23]. As thieno[2,3-*b*]pyridines function through polypharmacology (see below), unlike most current cancer drugs, they could be considered more difficult for tumor cells to develop resistance as many of their vital pathways are disrupted simultaneously. Compound **1** was also chosen as it was determined to generally have acceptable drug-like properties (Table S13).

After exposing HeLa and SiHa cell lines to compound **1** for 48 h, the EC_50_ values were found to be 2.14 µM and 2.77 µM, respectively. Several studies have explored the cytotoxic impact of thieno[2,3-*b*]pyridines on various cancer types, such as breast, prostate, and ovarian cancer [21,22,35,36]. Mastelić et al. showed that a compound similar to the one studied here had a cytotoxic impact on both breast and prostate cancer, though with a higher EC_50_ [36]. Additionally, in ovarian cancer, the EC_50_ value was approximately twice as high. This suggests that compound **1** might be more potent at lower concentrations [21].

Additionally, we demonstrated that compound **1** is non-toxic to normal cells, as over 90% of HEK-293 cells remained metabolically active after treatment with compound **1** across all doses and incubation times. Similar findings were observed previously in in vivo experiments. Two derivatives of thieno[2,3-*b*]pyridine were previously chosen for toxicity testing in mice at the National Cancer Institute’s Drug Therapeutic Programme [21,37]. The experiment involved administering intraperitoneal doses of 100, 200, and 400 mg/kg to three female athymic nude mice over a 20-day period for each compound. The survival of all mice throughout the treatment indicates that these compounds are well tolerated or safe even at high dosages.

The National Cancer Institute’s NCI60 panel of human cancer cell lines was used to evaluate nineteen compounds from the thieno[2,3-*b*]pyridine family [38]. Based on their effective growth inhibition at a concentration of 10 mM, compounds 1 through 5, along with compound 16 (6 derivates), were chosen for further evaluation in dose–response studies. The results showed notable effectiveness against five specific cancer types: melanoma (MDA-MB-435), breast cancer (MDA-MB-468), non-small cell lung cancer (NCI-H522), central nervous system cancer (SF-295), and leukemia (K-562). Additionally, several other cancer cell lines demonstrated positive responses to the compounds, including another breast cancer line (MDA-MB-468), an ovarian cancer line (OVCAR-3), and a renal cancer line (A498).

In another study, HCT-116 and MDA-MB-231 cells were treated with 1 µM of compound 1 exhibiting reduced mean relative growth compared to control cells, corresponding to 33.5% and 15.9%, respectively [23].

It has now been established that the thieno[2,3-*b*]pyridines modulate a number of biological targets related to tumorigenesis: (i) phospholipase C-δ1/3 (PLC), deduced by the same cellular behavior of the MDA-MB-231 breast cancer cell line upon administration of thieno[2,3-*b*]pyridines and following knock-down of the PLC-δ1/3 genes [39]; (ii) copper-trafficking antioxidant 1 (ATOX1) protein, the inhibition of which reduces the proliferation of cancer cells [40]; (iii) tyrosyl DNA phosphodiesterase 1 (TDP 1), a phospholipase D enzyme, involved in repairing DNA damage [41]; (iv) the colchicine binding site in tubulin [42,43], an established target for anticancer drugs; and (v) adenosine A_2A_ receptor (A2AAR) [44], a G protein-coupled receptor (GPCR). It can therefore be stated that the thieno[2,3-*b*]pyridines are multitargeting compounds and therefore function through polypharmacology [45].

The mechanism of cell death observed in the treated HeLa and SiHa cells with compound **1** was apoptosis. Apoptosis is an energy-dependent process that typically takes place without causing inflammation. Most apoptotic cells are removed by phagocytes while their cell membranes remain whole, preventing the release of potentially inflammatory intracellular contents. Late apoptotic cells, despite losing membrane integrity, still exhibit the characteristic behavior of early apoptotic cells [46]. Following treatment with compound **1**, there was a statistically significant increase in the percentage of early, late, and total apoptotic cells in the SiHa cell line compared to untreated cells. On the other hand, the HeLa cell line manifested a statistically significant increase in early and total apoptosis, but to a lesser extent. Gutiérrez et al. evaluated the effect of C6-ceramide on three cervical cancer cell lines, including HeLa, and discovered that C6 induces early and late apoptosis. However, the percentage of late apoptotic cells was significantly higher than the increase observed for early apoptosis [47]. Inducing pro-apoptotic mechanism suggests that compound **1** has the potential to reduce inflammation and be further investigated for its anticancer activity. Still, the difference between the two cervical cell lines may indicate that thieno[2,3-*b*]pyridines are able to induce different effects in different cervical cell lines. Considering our results, which show significant reduction in the number of HeLa cells treated with compound **1** in a dose-dependent manner, we can assume that compound **1** reduces HeLa cell proliferation and induces cell death (apoptosis) in SiHa cells.

Additionally, our findings of altered mitochondrial morphology following treatment in both cell lines suggest that the interrupted proliferation of HeLa cells and apoptosis in SiHa cells were likely driven by mitochondrial damage.

Cancer stem cells (CSCs) are cells possessing the ability for continuous self-renewal, which play a pivotal part in initiating tumor formation and accumulation of mutations in these cells may correlate with their capacity to promote resistance to anticancer therapies, leading to tumor progression and/or relapses [48,49]. After the 48 h treatment with compound **1**, we observed a reduction in the percentage of CSCs by 3.5 times in the SiHa cell line and 1.2 times in the HeLa cell line. This correlates with the finding of enhanced apoptosis rate in treated cells. Further investigation should provide us with a better understanding of the correlation between the mechanism of cell death in the cervical cell line and the percentage of CSCs. Conventional chemotherapy and radiotherapy primarily target differentiated cancer cells, leaving CSCs unaffected due to their resistance to these treatments [50]. The capacity of compound **1** to decrease the percentage of CSCs positions it as a promising drug capable of reducing tumor burden by overcoming resistance to therapy, as well as restraining distant metastasis and recurrence.

Glycosphingolipids (GSLs) are present in almost all cells and body fluids of vertebrates, with particularly high concentrations in the nervous system. Within cells, they are primarily, though not exclusively, localized on the plasma membrane [51]. Gangliosides, or acidic glycosphingolipids, are essential molecules involved in cellular recognition and signaling and are recognized for their role in regulating the majority of growth factor receptors [52]. They have also been linked to tumor growth and metastasis formation [53]. Kabayama et al. demonstrated when GM3 levels increase, it inhibits insulin signaling by binding to the insulin receptor (IR) and disrupting the interaction between caveolin-1 and the insulin receptor, thereby contributing to the development of insulin resistance [54]. GM3 can also inhibit EGFR without disrupting EGF binding [55]. Although compound **1** did not alter the percentage or expression of GM3 in CSCs or non-CSCs of either cell line, we spotted a statistically significant decrease in the expression and percentage of GM2 in non-CSCs, as well as a decrease in expression in CSCs, in the SiHa cell line. Miljan et al. showed that GM2 binds to EGFR, albeit with a 50% reduction compared to GM3. Additionally, they found that GM2 had a slight, though not statistically significant, effect in reducing EGFR autophosphorylation [56]. However, in addition to direct receptor binding, gangliosides may influence EGFR signaling pathways through various mechanisms. Yoshida et al. investigated the role of GD2 and GD3 in the phenotype of small-cell lung cancer cell lines. They concluded that increased expression of these gangliosides has been linked to accelerated cell growth and heightened invasiveness [57]. In our research, we demonstrated a decrease in the percentage of GD3 in CSCs of the SiHa cell line, which may relate to the effect of compound **1** in reducing the number of CSCs.

Regarding neutral GSLs, in the HeLa cell line, there was a statistically significant decrease in the expression of Gg_3_Cer in CSCs, and both the percentage and expression in non-CSCs. Pervan et al. demonstrated a similar effect with a related compound on the breast cancer cell line MCF-7 [35]. They attributed the decrease to the deletion of glycosyltransferase B4GALNT1, which is essential for Gg3Cer synthesis, and its knockdown has been manifested to cause a phenotype change from CSC to non-CSC [58]. Additionally, in the SiHa cell line, there was a statistically significant increase in the expression of nLc_4_Cer-positive cells in both CSCs and non-CSCs, as well as in the expression of ganglioside IV^3^Neu5Ac-nLc_4_Cer, which is its acidic version. However, in the HeLa cell line, the expression of IV^3^Neu5Ac-nLc_4_Cer was decreased in CSCs. In our previous study, we found a similar effect on two ovarian cancer cell lines [21].

Above all, our results of the impact of compound **1** on mitochondrial viability support the observed dysregulated GSL composition, as GSLs are well known to influence oxidative phosphorylation.

Metabolomic research is essential for identifying relevant metabolites and discovering biomarkers. It has been extensively studied for various aspects of cancer, including prevention, diagnosis, treatment, and prediction of disease progression [59]. Metabolic biomarkers for cervical cancer offer insights into the progression and severity of the disease. Regarding the metabolic profiling of the two cervical cancer cell lines, HeLa and SiHa, we observed statistically significant different effects after treatment with compound **1**. In the HeLa cell line, treatment with compound **1** resulted in a 2-fold increase in glycerol levels.

It is well established that cancer and chronic diseases are linked. Many organisms metabolize glycerol by transforming it into intermediate metabolites that are essential for creating vital cellular components, such as lipids. This metabolic process begins with the conversion of glycerol to glycerol-3-phosphate (G3P), a crucial precursor in lipid biosynthesis [60]. Modifying glycerol metabolism leads to changes in the final lipid content [60]. Triacylglycerol (TAG), a fatty acid ester derivate of glycerol, serves as the main energy storage unit in eukaryotic cells and is particularly active in adipocytes [61,62]. Similarly, improper accumulation of neutral lipids is associated with numerous major diseases, including obesity and diabetes. In their research, Marin et al. investigated the potential role of modified lipid profiles and chronic hyperglycemia in the development of endometrial cancer. They demonstrated that patients with endometrial cancer show a tendency toward higher levels of triglycerides and glycated hemoglobin, as well as an increased body mass index [63]. Rosato et al. pointed out a significant correlation between the number of metabolic syndrome components and the risk of endometrial cancer, especially overweight, but also diabetes, hypertension, and hyperlipidemia [64]. In cervical cancer, Shen et al. found that elevated blood sugar levels increased the risk of stromal invasion. However, they noted that obesity was negatively correlated with lymph node metastasis [65]. On the other hand, in their latest systematic review, they stated that patients with a body mass index higher than 35 were at a significantly higher risk of lymph node metastasis, although without a statistically significant difference [66].

A recent study has shown the localization of TAG to the site of cell division, suggesting that TAGs can also associate with membranes and play roles in cellular processes beyond energy storage [67,68]. Li et al. demonstrated that during apoptosis, TAGs containing polyunsaturated fatty acyl chains (PUFA-TAGs) accumulate and are stored in lipid droplets, regardless of the cytotoxic agents and cell lines used [68]. They pointed out that storing polyunsaturated fatty acids as PUFA-TAGs in lipid droplets may preserve cells from lipid peroxide-induced membrane damage during heightened oxidative stress, limiting cell death during apoptosis. This might account for the lower percentage of apoptotic cells in the HeLa cell line, as well as the reduction in the percentage of CSCs.

Pappa et al. demonstrated in their study that HPV-positive HeLa and SiHa cells exhibited a preference for accumulating glucose and glycolytic intermediates like glucose-6-phosphate, fructose-6-phosphate, and lactate. These alterations are accompanied by a relative depletion of pyruvate, indicating the activation of aerobic glycolysis (Warburg metabolism) in these cells [69]. Compound **1** enlarged glucose and lactate acid levels in the SiHa cell line, though this was not statistically significant, which correlates with their finding. The increase in these two metabolites further supports our findings regarding the negative impact of compound **1** on mitochondrial viability and number. However, treatment with compound **1** resulted in a statistically significant decrease in octadecanol levels in the SiHa cell line. According to the Human Metabolome Database, octadecanol is a fatty alcohol found in humans and is typically incorporated into plasmalogen lipids which participate in cell signaling, fatty acid metabolism, lipid peroxidation, and plasmalogen synthesis. Its biological role is to stabilize membranes. Braverman et al. demonstrated in their article that plasmalogens protect membrane lipids from oxidation and facilitate signaling processes. However, their functions are likely specific to the tissue type and developmental stage [70]. From these results, we can conclude that compound **1** compromises membrane integrity and leads cells towards death.

## 4. Materials and Methods

### 4.1. Cell Culture and Thieno[2,3-b]pyridine Compound

Human embryonic kidney cells (HEK.293) and cervical cancer cell lines HeLa and SiHa (HTB-35), purchased from ATCC (American Type Culture Collection, LGC Standards, Teddington, Middlesex, UK), were grown in Dulbecco’s Modified Eagle Medium (HEK-293 and HeLa, DMEM, with L-glutamine, Sigma-Aldrich, Steinheim, Germany) and Eagle’s Minimum Essential Medium (SiHa, EMEM, Sigma-Aldrich, Steinheim, Germany), respectively, with the addition of 10% fetal bovine serum (FBS, EuroClone, Milan, Italy) and 1% antibiotics (penicillin/streptomycin, Sigma-Aldrich, Steinheim, Germany) in an incubator at 37 °C and 5% CO_2_.

Cells from all three cell lines, HEK-293, HeLa and SiHa, were treated with four different cytotoxic thieno[2,3-*b*]pyridines. These compounds were discovered by virtual high throughput screens (vHTS) as potential inhibitors of phospholipase C isoforms, by Reynisson et al. [20]. Four different compounds were then dissolved in dimethyl sulfoxide (DMSO) to calculate the half-maximal effective concentration (EC_50_). As a result of maximum effectiveness, (*E*)-3-amino-5-(3-(3-bromophenyl)acryloyl)-*N*-(3-chloro-2-methylphenyl)-6-methylthieno[2,3-*b*]pyridine-2-carboxamide (compound **1**) was chosen for this study.

### 4.2. Measurement of Cytotoxic Activity

To determine the percentage of metabolically active normal (HEK-293) and cervical cancer cells (HeLa and SiHa), the 3-(4,5-dimethylthiazol-2-yl)-2,5-diphenyltetrazole bromide (MTT) test was performed [71]. An equal number of cells (1 × 10^4^) were seeded in triplicates on 96-well microtiter plates and incubated overnight. The next day, the cervical cancer cells were treated with medium (control cells) and solutions of different concentrations of the four thieno[2,3-*b*]pyridines (0.05 µM, 0.2 µM, 0.5 µM, 1 µM, 2.5 µM, 5 µM and 10 µM) in the medium and left to incubate for 4, 24, 48 and 72 h. After this treatment, the cells were incubated with 0.5 mg/mL MTT solution for 2 h. The solution was then removed and DMSO was added, after which the absorbance at 570 nm was measured using a HiPo MPP-96 microtiter plate reader (Biosan, Riga, Latvia).

The same procedure was performed on normal HEK-293 cells, but the treatment included only compound **1** (treated cells) and complete medium (control cells).

### 4.3. Flow Cytometric Analyses

#### 4.3.1. Apoptosis

An equal number of cells (1 × 10^5^) were seeded in triplicates on 6-well microtiter plates and treated with EC_50_ concentration of compound **1**: 2.5 µM for HeLa cell line and 2.14 µM for SiHa cell line. After 48 h treatment with compound **1**, the cells were trypsinized, washed with phosphate buffer solution (PBS) and resuspended in 100 μL of binding buffer containing 5 μL of Annexin-V-fluorescein isothiocyanate (FITC) and 10 μL of propidium iodide (PI) (FITC Annexin V Apoptosis Detection Kit with PI, BioLegend, San Diego, CA, USA). After 15 min of incubation at room temperature in the dark, the cells were analyzed by flow cytometry (BD Accuri C6, BD Biosciences, Franklin Lakes, NJ, USA). The percentage of apoptotic cells and standard deviation were analyzed by the FlowLogic program (Inivai, Victoria, Australia).

#### 4.3.2. Glycosphingolipid (GSL) Expression

An equal number of cells (1 × 10^5^) were seeded in 6-well plates and treated with 2.14 µM of compound **1**, for HeLa cell line, and 2.77 µM of compound **1** for SiHa cell line for 48 h and then trypsinized and washed with PBS. The cells were incubated with antibodies for stem cells and GSL markers. Samples from both cell lines were stained with anti-CD49f-FITC (Rat Anti-Human CD49f-FITC, BD Pharmingen, Franklin Lakes, NJ, USA), anti-ALDH1A1-PE (ab209437, Abcam, Cambridge, UK) and anti-GSL antibodies. The primary antibody against GD3 (mouse IgG3) was produced by laboratory of Dr. J. Müthing and antibody against GM3 (mouse IgM) was from Cosmo Bio Co. (Tokyo, Japan). The primary antibodies against all other GSLs (Gb_4_Cer, nLc_4_Cer, IV^3^Neu5Ac-nLc_4_Cer, GM2, Gg_3_Cer and GalNAc-GM1b) were chicken polyclonal antibodies, being produced and characterized by the laboratory of Dr. J. Müthing [72]. The detection of primary anti-GSL antibody binding was accomplished using secondary antibodies conjugated with eFluor 660 fluorochrome (Abcam, Cambridge, UK). Stained sample data acquisition was conducted using a BD Accuri 6 cytometer (BD Biosciences, San Diego, CA, USA) and afterwards analyzed with the FlowLogic Software 8.1 (Inivai, Melbourne, VIC, Australia).

#### 4.3.3. Tracking of Mitochondria

An equal number of cells (1 × 10^5^) were seeded in 6-well plates and treated with 2.14 µM of compound **1**, for HeLa cell line, and 2.77 µM of compound **1** for SiHa cell line for 48 h and then detached using trypsin, followed by washing twice with PBS + 5% FBS. The cells were then incubated in 500 μL of 100 nM solution of MitroTracker Deep Red FM (M22426, Thermo Fisher Scientific, Walthman, MA, USA) for 30 min in dark at 37 °C. Immediately before flow cytometry, 1 μL of propidium iodide (PI) (BioLegend, San Diego, CA, USA) was added, to detect dead cells. Sample acquisition was performed using a BD Accuri 6 cytometer (BD Biosciences, San Diego, CA, USA) and analyzed with the FlowLogic Software.

### 4.4. Statistical Analysis

The EC_50_ values and statistical analysis of cytotoxic effects were computed using GraphPad Prism 7.0 (San Diego, CA, USA). Statistical analyses for apoptosis and GSL expression included *t*-tests with unequal variances, one-way ANOVA followed by post hoc Tukey tests, or Kruskal–Wallis tests followed by Dunn’s post hoc tests. These analyses were conducted using the same statistical software GraphPad Prism 7.0, with significance set at *p*-value < 0.05.

### 4.5. Metabolite Analyses

#### 4.5.1. Sample Collection

Cells were grown for 48 h in 6-well plates to near confluence. The culture medium was discarded, and cells were gently washed with 2 mL PBS. PBS was removed, and 1.5 mL of methanol was added. Then, 10 μL of ribitol (Sigma Aldrich, Steinheim, Germany) was added as an internal standard. Collected supernatants were evaporated under the nitrogen blowdown.

#### 4.5.2. Sample Derivatization and GC-MS Analysis

The derivatization procedure included the addition of 25 μL solution consisting of 20 mg/mL metoxylamine hydrochloride in pyridine. Afterward, continuous shaking was performed for 90 min at 30 °C followed by addition of MSTFA (*N*-methyl-*N*-(trimethylsilyl)trifluoroacetamide, Sigma Aldrich, Steinheim, Germany) + 1% TMCS (trimethylchlorosilane, Sigma Aldrich, Steinheim, Germany) with incubation at 50 °C for 30 min for complete derivatization. The samples were dissolved in 100 μL pyridine.

The samples underwent analysis employing an Agilent 8890 GC system coupled with triple quad spectrometer system MS 7000D GC/TQ. The column was HP-5 MS (30 m × 0.25 mm × 0.25 μm, Agilent, Santa Clara, CA, USA) with an oven program set on 60 °C held for 2 min, then increased to 210 °C at a rate of 10 °C/min, ramped to 240 °C at a rate of 5 °C/min, ramped to 315 °C at a rate of 25 °C/min and then maintained at 315 °C for 3 min.

#### 4.5.3. GC-MS Data Pre-Processing and Statistical Analysis

Agilent Mass Hunter Qualitative Analysis software version 10.0 was used for data processing. Mass spectra and peaks were compared with NIST mass spectral library (Wiley, Hoboken, NJ, USA). The intensity value for every metabolite was adjusted based on the signal from the ribitol internal standard. Statistical analysis was performed using a series of Workflows created in the Galaxy web-based platform.

MetaboAnalyst, a specialized platform for metabolomics data analysis, was used to establish a panel of differentially expressed metabolites across these two cervical cell lines. A series of *t*-tests were performed for statistical significance analysis. The effect of compound **1** on both cell lines was compared using one-way ANOVA. Principal component analysis (PCA) was used as an unsupervised clustering method. Metabolic set enrichment analysis (MSEA) was utilized to establish a connection between metabolic fingerprints and changes in metabolite concentrations.

### 4.6. Fluorescence Microscopy

The cells were seeded on glass coverslips and treated with 2.14 µM of compound **1**, for HeLa cell line, and 2.77 µM of compound **1** for SiHa cell line for 48 h. After treatment, cells were washed twice with PBS + 5% FBS and then incubated in 100 nM solution of MitroTracker Green FM (M7514, Thermo Fisher Scientific) for 30 min in dark at 37 °C. Coverslips were mounted to slide using Mowiol 4-88 Reagent (476904, Merck, Darmstadt, Germany) with DAPI (D9542, Merck, Darmstadt, Germany). Microscopy was performed on Olympus BX43 microscope and figures analyzed with ImageJ software version 1.54k.

## 5. Conclusions

Our study documented for the first time the effects of the thieno[2,3-*b*]pyridine anticancer compound, (*E*)-3-amino-5-(3-3-bromophenyl)acryloyl)-*N*-(3-chloro-2-methylphenyl)-6-methylthieno[2,3-*b*]pyridine-2-carboxamide (compound **1**), on cervical cancer. We verified our hypothesis that it would exhibit cytotoxic effects, specifically showing a preference for inducing apoptosis. However, the variation between the two cervical cell lines suggests that thieno[2,3-*b*]pyridine might trigger different cell death mechanisms in each line. Moreover, the percentage of CSCs was significantly reduced. This insight could be applied to the development of compound **1** as an antitumor medication, especially when considering the role of CSCs in developing resistance to anticancer therapies, which leads to tumor progression and/or relapses.

Previous studies have demonstrated the use of metabolomics for detecting cervical cancer biomarkers. Our study thoroughly examined the alterations in GSLs and metabolites after treatment with compound **1**. In summary, the study emphasized the crucial need for discovering a new therapy, and compound **1** warrants further investigation in both in vitro and in vivo models.

## Figures and Tables

**Table 1:** List of identified metabolites in the HeLa and SiHa cell lines after 48 h treatment with compound **1**.

No.	Metabolite	HeLa		SiHa	
		*p*-Value	Fold Change	*p*-Value	Fold Change
1	Fructose	0.0933	8.6667	0.1157	0.36235
2	Erythritol	0.1157	2.5974	-	-
3	**Glycerol**	0.0439 *	**2.177**	-	-
4	Myristic acid	0.3537	2.0952	0.2924	0.68703
5	Nonadecane	0.7063	1.3947	-	-
6	1-Acylglycerol	0.4000	1.2654	0.1458	0.51757
7	2-Stearoylglycerophosphoglycerol	0.8888	1.2517	1.000	0.79479
8	Xylose	0.3941	1.213	-	-
9	Stearic acid	0.2794	1.1812	0.4050	0.71185
10	**Octadecanol**	0.1409	1.172	0.0003 *	**0.7916**
11	Pyruvic acid	0.2835	1.1719	-	-
12	Butyric acid	0.8099	1.1667	-	-
13	Myo inositol	0.6579	1.1543	0.5506	1.0463
14	Behenic acid	0.1401	1.1304	0.1642	0.70783
15	Dibutyl phthalate	0.2612	1.1286	0.9625	0.99585
16	Palmitic acid	0.1696	1.1197	0.1150	1.2907
17	Lactic acid	0.5214	0.91443	0.8880	1.0147
18	Mannitol	0.6779	1.0656	-	-
19	Galactose	0.6995	1.0627	0.0953	1.1795
20	Succinic acid	0.4778	1.0625	-	-
21	Cholesterol	0.7865	0.94372	0.1637	0.6011
22	L-Proline	0.6972	1.0479	0.1044	1.4
23	Phosphate	0.7821	1.0199	0.2566	1.2162
24	Glucose	-	-	0.1841	1.336
25	Oleic acid	-	-	0.6295	1.3
26	Inositol	-	-	1.000	0.79592
27	L-Glutamic acid	-	-	0.7788	0.84283
28	3-Hydroxybutyric acid	-	-	0.7458	1.064
29	Hydrocinnamic acid	-	-	0.8995	0.92891

Notes: The fold change indicates the ratio of the mean signal intensity values (across three independent experiments) of treated cells compared to untreated cells. *p*-values were determined using the Student *t*-test (entries with over 50% missing values were excluded). * statistically significant difference.

## Data Availability

The data presented in this study are available on request from the corresponding author.

## Supplementary Materials

The following supporting information can be downloaded at: https://www.mdpi.com/article/10.3390/ijms26062651/s1.
