# Supplementary material for: Thieno[2,3-b]pyridines as a Novel Strategy Against Cervical Cancer: Mechanistic Insights and Therapeutic Potential
Source: Int J Mol Sci. 2025 Mar 14;26(6):2651. doi: 10.3390/ijms26062651 (PMC11942470; doi:10.3390/ijms26062651)
Supplement: Supplementary file 1 [file ijms-26-02651-s001.zip › ijms-3482049-supplementary.pdf]

Supplementary Files

Cervical cancer and thieno[2,3-*b*]pyridines – a step closer to the entity we leave behind?

Monika Čikeš Botić, Sandra Marijan, Mila Radan, Lisa I. Pilkington, Zdravko Odak, David Barker, Jóhannes Reynisson and Vedrana Čikeš Čulić

Index

Figure S1. 3-amino-*N*-(3-chloro-2-methylphenyl)-5-oxo-5,6,7,8-tetrahydrothieno[2,3-*b*]quinoline-2-carboxamide…………..3

Table S1. The effect of 3-amino-*N*-(3-chloro-2-methylphenyl)-5-oxo-5,6,7,8-tetrahydrothieno[2,3-*b*]quinoline-2-carboxamide on HeLa cell line; Cells were treated with 7 different concentrations for 4, 24, 48 and 72 h………….3

Figure S2. Cell viability of HeLa cell line after treatment with 3-amino-*N*-(3-chloro-2-methylphenyl)-5-oxo-5,6,7,8-tetrahydrothieno[2,3-*b*]quinoline-2-carboxamide…………...4

Table S2. EC_{50} after 4, 24, 48 and 74 h after treatment with 3-amino-*N*-(3-chloro-2-methylphenyl)-5-oxo-5,6,7,8-tetrahydrothieno[2,3-*b*]quinoline-2-carboxamide on HeLa cell line…………4

Table S3. The effect of 3-amino-*N*-(3-chloro-2-methylphenyl)-5-oxo-5,6,7,8-tetrahydrothieno[2,3-*b*]quinoline-2-carboxamide on SiHa cell line, Cells were treated with 7 different concentrations for 24, 48 and 72 h…………..5

Figure S3. Cell viability of SiHa cell line after treatment with 3-amino-*N*-(3-chloro-2-methylphenyl)-5-oxo-5,6,7,8-tetrahydrothieno[2,3-*b*]quinoline-2-carboxamide…………...5

Table S4. EC_{50} after 24, 48 and 74 h after treatment with 3-amino-*N*-(3-chloro-2-methylphenyl)-5-oxo-5,6,7,8-tetrahydrothieno[2,3-*b*]quinoline-2-carboxamide on SiHa cell line…………6

Figure S4. 3-amino-*N*-(naphthalen-1-yl)-5-oxo-5,6,7,8-tetrahydrothieno[2,3-*b*]quinoline-2-carboxamide....7

Table S5. The effect of 3-amino-*N*-(naphthalen-1-yl)-5-oxo-5,6,7,8-tetrahydrothieno[2,3-*b*]quinoline-2-carboxamide on HeLa cell line; Cells were treated with 7 different concentrations for 4, 24, 48 and 72 h….7

Figure S5. Cell viability of HeLa cell line after treatment with 3-amino-*N*-(naphthalen-1-yl)-5-oxo-5,6,7,8-tetrahydrothieno[2,3-*b*]quinoline-2-carboxamide…………...8

Table S6. EC_{50} after 4, 24, 48 and 74 h after treatment with 3-amino-*N*-(naphthalen-1-yl)-5-oxo-5,6,7,8-tetrahydrothieno[2,3-*b*]quinoline-2-carboxamide on HeLa cell line………….8

Table S7. The effect of 3-amino-*N*-(naphthalen-1-yl)-5-oxo-5,6,7,8-tetrahydrothieno[2,3-*b*]quinoline-2-carboxamide on SiHa cell line; Cells were treated with 7 different concentrations for 24, 48 and 72 h……..9

Figure S6. Cell viability of SiHa cell line after treatment with 3-amino-*N*-(naphthalen-1-yl)-5-oxo-5,6,7,8-tetrahydrothieno[2,3-*b*]quinoline-2-carboxamide……………………………………………………………..9

Table S8. EC_{50} after 24, 48 and 74 h after treatment with 3-amino-*N*-(naphthalen-1-yl)-5-oxo-5,6,7,8-tetrahydrothieno[2,3-*b*]quinoline-2-carboxamide on SiHa cell line……………………………….…...10

Table S9. The effect of Compound 1 on HeLa cell line; Cells were treated with 7 different concentrations of the Compound 1 for 4, 24, 48 and 72 h…………………………………………………………………...11

Table S10. EC_{50} after 4, 24, 48 and 74 h after treatment with Compound 1 on HeLa cell line………………………………………………………………………………………………………11

Table S11. The effect of Compound 1 on SiHa cell line; Cells were treated with 7 different concentrations of the Compound 1 for 4, 24, 48 and 72 h…………………………………………………………………...12

Table S12. EC_{50} after 4, 24, 48 and 74 h after treatment with Compound 1 on SiHa cell line……………......12

Figure S7. Cancer stem cells (ALDH+) after 48 h treatment with compound **1** on HeLa and SiHa cell lines …………………………………………………………………………………………………...13

Table S13. Calculated physiochemical properties of Compound 1 as determined by ADMET RankerTM……………………………………………………………………………………….……...…13

Figure S8. (E)-3-amino-5-(3-(3-bromophenyl)-1-hydroxyallyl)-*N*-(3-chloro-2-methylphenyl)-6-methylthieno[2,3-*b*]quinoline-2-carboxamide…………………………………………………………….....14

Table S14. The effect of (E)-3-amino-5-(3-(3-bromophenyl)-1-hydroxyallyl)-*N*-(3-chloro-2-methylphenyl)-6-methylthieno[2,3-*b*]quinoline-2-carboxamide on HeLa cell line; Cells were treated with 7 different concentrations of the for 4, 24, 48 and 72 h…………………………………………………………..…...14

Figure S9. Cell viability of HeLa cell line after treatment with (E)-3-amino-5-(3-(3-bromophenyl)-1-hydroxyallyl)-*N*-(3-chloro-2-methylphenyl)-6-methylthieno[2,3-*b*]quinoline-2-carboxamide………..…….15

Table S15. EC_{50} after 4, 24, 48 and 74 h after treatment with (E)-3-amino-5-(3-(3-bromophenyl)-1-hydroxyallyl)-*N*-(3-chloro-2-methylphenyl)-6-methylthieno[2,3-*b*]quinoline-2-carboxamide on HeLa cell line………………………………………………………………………………………...............15

Table S16. The effect of (E)-3-amino-5-(3-(3-bromophenyl)-1-hydroxyallyl)-*N*-(3-chloro-2-methylphenyl)-6-methylthieno[2,3-*b*]quinoline-2-carboxamide on SiHa cell line; Cells were treated with 7 different concentrations for 24, 48 and 72 h……………………………………………………………..………..16

Figure S10. Cell viability of SiHa cell line after treatment with (E)-3-amino-5-(3-(3-bromophenyl)-1-hydroxyallyl)-*N*-(3-chloro-2-methylphenyl)-6-methylthieno[2,3-*b*]quinoline-2-carboxamide………..…….16

Table S17. EC_{50} after 24, 48 and 74 h after treatment with (E)-3-amino-5-(3-(3-bromophenyl)-1-hydroxyallyl)-*N*-(3-chloro-2-methylphenyl)-6-methylthieno[2,3-*b*]quinoline-2-carboxamide on SiHa cell line…………………………………………………………………………………………..……..17

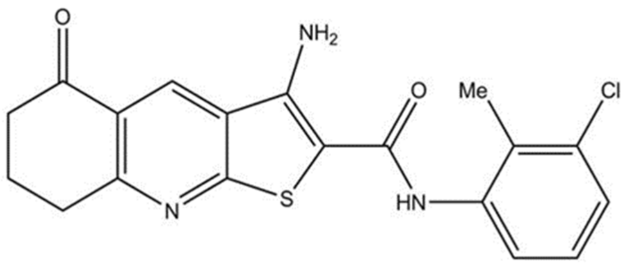

Figure S1. 3-amino-*N*-(3-chloro-2-methylphenyl)-5-oxo-5,6,7,8-tetrahydrothieno[2,3-*b*]quinoline-2-carboxamide.

Table S1. The effect of 3-amino-*N*-(3-chloro-2-methylphenyl)-5-oxo-5,6,7,8-tetrahydrothieno[2,3-*b*]quinoline-2-carboxamide on HeLa cell line

Cells were treated with 7 different concentrations for 4, 24, 48 and 72 h.

	p-value			
	4 h	24 h	48 h	72 h
0.05 μM	0.079	0.025	0.021*	0.001**
0.2 μM	0.531	0.057	0.003**	<0.001***
0.5 μM	0.658	0.655	<0.001***	<0.001***
1 μM	0.955	0.288	<0.001***	<0.001***
2.5 μM	0.078	0.018	<0.001***	<0.001***
5 μM	0.019	0.065	<0.001***	<0.001***
10 μM	0.468	0.033	<0.001***	<0.001***

Note: p-values relate to a two-sample t-test comparing the treatment at the given concentration with when no treatment is applied (0 μM, control); * p-value < 0.05; ** p-value < 0.01; *** p-value < 0.001.

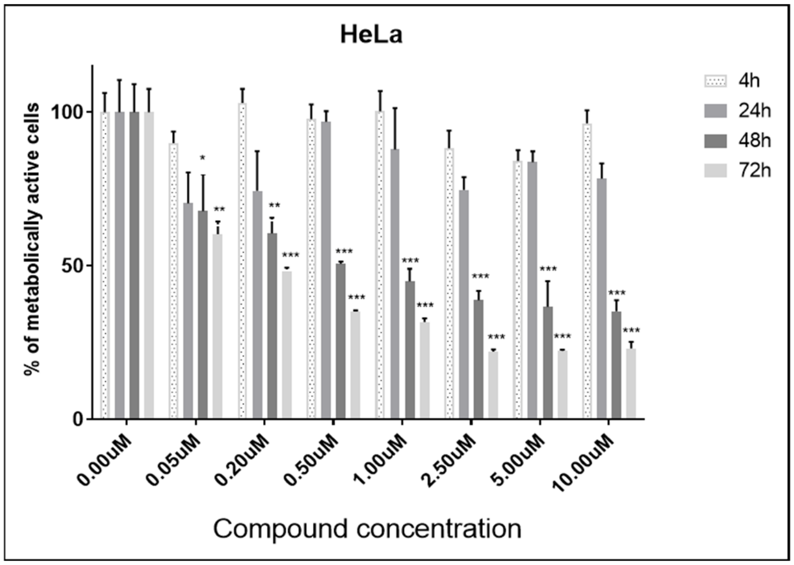

Figure S2. Cell viability of HeLa cell line after treatment with 3-amino-*N*-(3-chloro-2-methylphenyl)-5-oxo-5,6,7,8-tetrahydrothieno[2,3-*b*]quinoline-2-carboxamide. *Notes:* Data are expressed as a mean from the experiment performed in triplicate ± SD. Columns, mean of metabolically active cells; bars, SD (standard deviation); * p-value < 0.05; ** p-value < 0.01; *** p-value < 0.001 (p-values relate to a two-sample t-test comparing the treatment at the given concentration with when no treatment is applied (0 μM).

Table S2. EC_{50} after 4, 24, 48 and 74 h after treatment with 3-amino-*N*-(3-chloro-2-methylphenyl)-5-oxo-5,6,7,8-tetrahydrothieno[2,3-*b*]quinoline-2-carboxamide on HeLa cell line.

	4 h	24 h	48 h	72 h
EC_{50} / μM	ND	ND	0.8241	0.2449

Note: ND, could not be determined.

Table S3. The effect of 3-amino-*N*-(3-chloro-2-methylphenyl)-5-oxo-5,6,7,8-tetrahydrothieno[2,3-*b*]quinoline-2-carboxamide on SiHa cell line

Cells were treated with 7 different concentrations for 24, 48 and 72 h.

	p-value		
	24 h	48 h	72 h
0.05 μM	0.341	0.523	0.013*
0.2 μM	0.940	0.002**	<0.001***
0.5 μM	0.044	<0.001***	<0.001***
1 μM	0.129	<0.001***	0.019*
2.5 μM	0.014	<0.001***	<0.001***
5 μM	0.011	<0.001***	<0.001***
10 μM	0.005	<0.001***	<0.001***

Note: p-values relate to a two-sample t-test comparing the treatment at the given concentration with when no treatment is applied (0 μM, control); * p-value < 0.05; ** p-value < 0.01; *** p-value < 0.001.

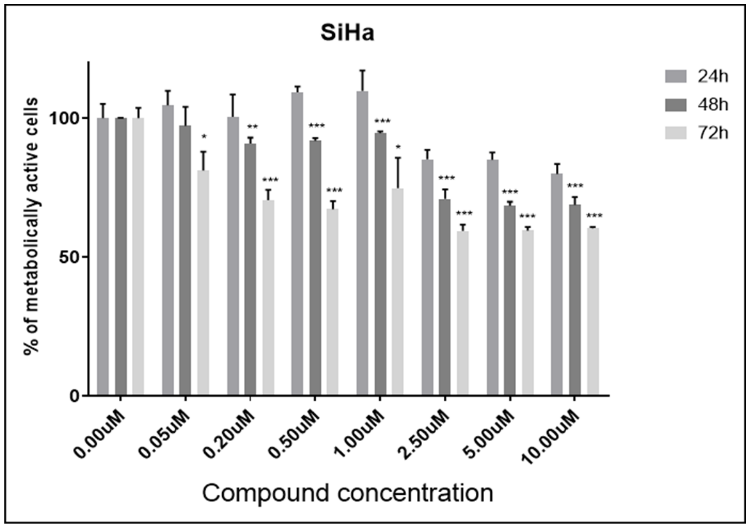

Figure S3. Cell viability of SiHa cell line after treatment with 3-amino-*N*-(3-chloro-2-methylphenyl)-5-oxo-5,6,7,8-tetrahydrothieno[2,3-*b*]quinoline-2-carboxamide. *Notes:* Data are expressed as a mean from the experiment performed in triplicate ± SD. Columns, mean of metabolically active cells; bars, SD (standard deviation); * p-value < 0.05; ** p-value < 0.01; *** p-value < 0.001 (p-values relate to a two-sample t-test comparing the treatment at the given concentration with when no treatment is applied (0 μM).

Table S4. EC_{50} after 24, 48 and 74 h after treatment with 3-amino-*N*-(3-chloro-2-methylphenyl)-5-oxo-5,6,7,8-tetrahydrothieno[2,3-*b*]quinoline-2-carboxamide on SiHa cell line.

	24 h	48 h	72 h
EC_{50} / μM	ND	ND	6.104

Note: ND, could not be determined.

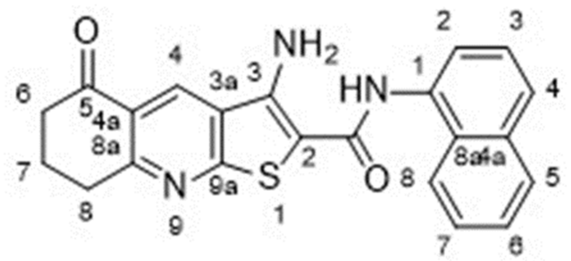

Figure S4. 3-amino-*N*-(naphthalen-1-yl)-5-oxo-5,6,7,8-tetrahydrothieno[2,3-*b*]quinoline-2-carboxamide.

Table S5. The effect of 3-amino-*N*-(naphthalen-1-yl)-5-oxo-5,6,7,8-tetrahydrothieno[2,3-*b*]quinoline-2-carboxamide on HeLa cell line

Cells were treated with 7 different concentrations for 4, 24, 48 and 72 h.

	p-value			
	4 h	24 h	48 h	72 h
0.05 μM	0.049	0.117	0.109	0.044
0.2 μM	0.041	0.1678	0.008	0.091
0.5 μM	0.684	0.164	0.014	0.975
1 μM	0.417	0.306	0.045	0.039
2.5 μM	0.200	0.267	0.045	0.212
5 μM	0.571	0.124	0.019	0.024
10 μM	0.566	0.028	0.002	0.001**

Note: p-values relate to a two-sample t-test comparing the treatment at the given concentration with when no treatment is applied (0 μM, control); ** p-value < 0.01.

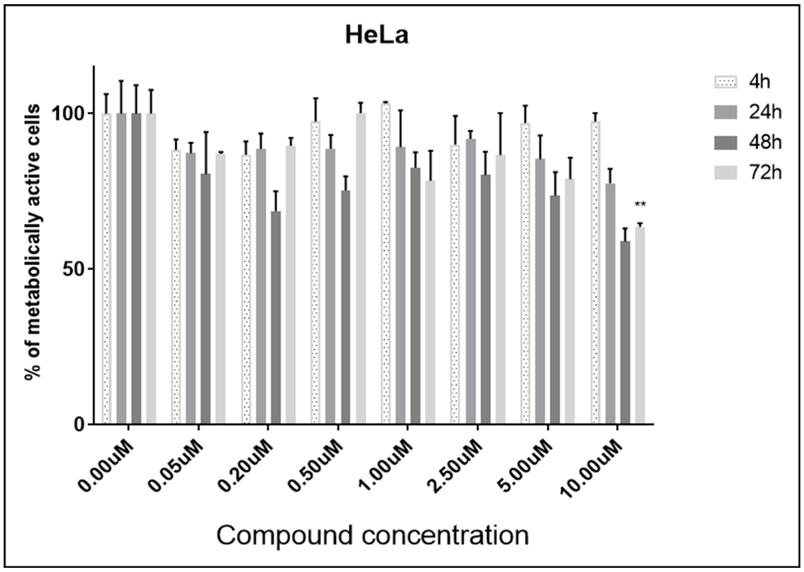

Figure S5. Cell viability of HeLa cell line after treatment with 3-amino-*N*-(naphthalen-1-yl)-5-oxo-5,6,7,8-tetrahydrothieno[2,3-*b*]quinoline-2-carboxamide. *Notes:* Data are expressed as a mean from the experiment performed in triplicate ± SD. Columns, mean of metabolically active cells; bars, SD (standard deviation); ** p-value < 0.01 (p-value relate to a two-sample t-test comparing the treatment at the given concentration with when no treatment is applied (0 μM).

Table S6. EC_{50} after 4, 24, 48 and 74 h after treatment with 3-amino-*N*-(naphthalen-1-yl)-5-oxo-5,6,7,8-tetrahydrothieno[2,3-*b*]quinoline-2-carboxamide on HeLa cell line.

	4 h	24 h	48 h	72 h
EC_{50} / μM	ND	ND	ND	ND

Note: ND, could not be determined.

Table S7. The effect of 3-amino-*N*-(naphthalen-1-yl)-5-oxo-5,6,7,8-tetrahydrothieno[2,3-*b*]quinoline-2-carboxamide on SiHa cell line

Cells were treated with 7 different concentrations for 24, 48 and 72 h.

	p-value		
	24 h	48 h	72 h
0.05 μM	0.088	<0.001***	0.031*
0.2 μM	0.315	<0.001***	0.002**
0.5 μM	0.016	0.005**	0.002**
1 μM	0.022	<0.001***	0.011*
2.5 μM	0.413	<0.001***	0.001**
5 μM	0.279	0.006**	0.001**
10 μM	0.500	0.018*	0.003**

Note: p-values relate to a two-sample t-test comparing the treatment at the given concentration with when no treatment is applied (0 μM, control); * p-value < 0.05; ** p-value < 0.01; *** p-value < 0.001.

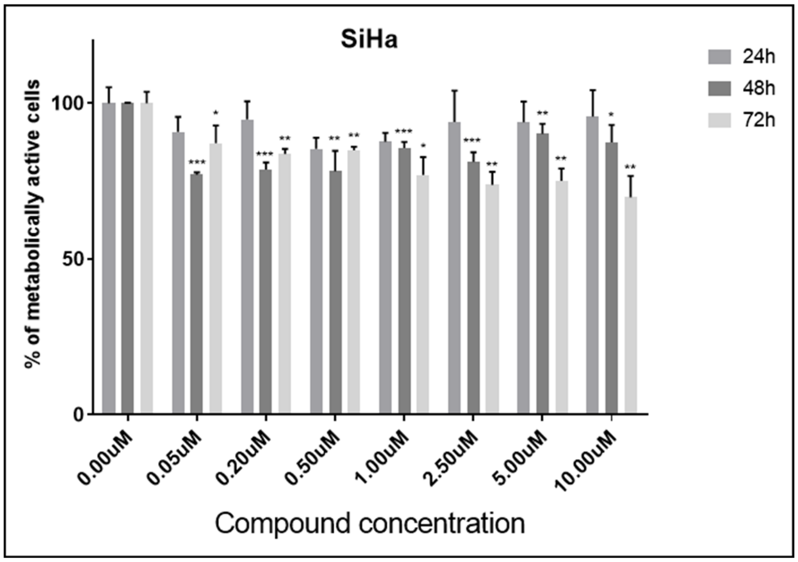

Figure S6. Cell viability of SiHa cell line after treatment with 3-amino-*N*-(naphthalen-1-yl)-5-oxo-5,6,7,8-tetrahydrothieno[2,3-*b*]quinoline-2-carboxamide. *Notes:* Data are expressed as a mean from the experiment performed in triplicate ± SD. Columns, mean of metabolically active cells; bars, SD (standard deviation); * p-value < 0.05; ** p-value < 0.01; *** p-value < 0.001 (p-values relate to a two-sample t-test comparing the treatment at the given concentration with when no treatment is applied (0 μM).

Table S8. EC_{50} after 24, 48 and 74 h after treatment with 3-amino-*N*-(naphthalen-1-yl)-5-oxo-5,6,7,8-tetrahydrothieno[2,3-*b*]quinoline-2-carboxamide on SiHa cell line.

	24 h	48 h	72 h
EC_{50} / μM	ND	ND	ND

Note: ND, could not be determined.

Table S9. The effect of compound **1** on HeLa cell line

Cells were treated with 7 different concentrations of the compound **1** for 4, 24, 48 and 72 h.

	p-value			
	4 h	24 h	48 h	72 h
0.05 μM	0.109	0.016	0.003**	0.034
0.2 μM	0.394	0.074	0.004**	Not determined
0.5 μM	0.002	0.060	Not determined	0.003**
1 μM	0.019	0.057	0.040*	0.011*
2.5 μM	0.069	0.005	<0.001***	0.001**
5 μM	0.015	0.003	<0.001***	<0.001***
10 μM	0.016	0.002	<0.001***	<0.001***

Note: p-values relate to a two-sample t-test comparing the treatment at the given concentration with when no treatment is applied (0 μM, control); * p-value < 0.05; ** p-value < 0.01; *** p-value < 0.001.

Table S10. EC_{50} after 4, 24, 48 and 74 h after treatment with compound **1** on HeLa cell line.

	4 h	24 h	48 h	72 h
EC_{50} / μM	ND	6.296	2.138	2.115

Note: ND, could not be determined.

Table S11. The effect of compound 1 on SiHa cell line

Cells were treated with 7 different concentrations of the compound 1 for 4, 24, 48 and 72 h.

	p-value			
	4 h	24 h	48 h	72 h
0.05 μM	<0.001***	0.002**	<0.001***	0.002**
0.2 μM	<0.001***	0.003**	0.002**	0.002**
0.5 μM	<0.001***	0.003**	0.014*	0.006**
1 μM	<0.001***	0.005**	<0.001***	0.014*
2.5 μM	<0.001***	0.001**	<0.001***	<0.001*
5 μM	<0.001***	<0.001***	<0.001***	<0.001***
10 μM	<0.001***	0.003**	<0.001***	<0.001***

Note: p-values relate to a two-sample t-test comparing the treatment at the given concentration with when no treatment is applied (0 μM, control); * p-value < 0.05; ** p-value < 0.01; *** p-value < 0.001.

Table S12. EC_{50} after 4, 24, 48 and 74 h after treatment with compound 1 on SiHa cell line.

	4 h	24 h	48 h	72 h
EC_{50} / μM	ND	ND	2.906	4.122

Note: ND, could not be determined.

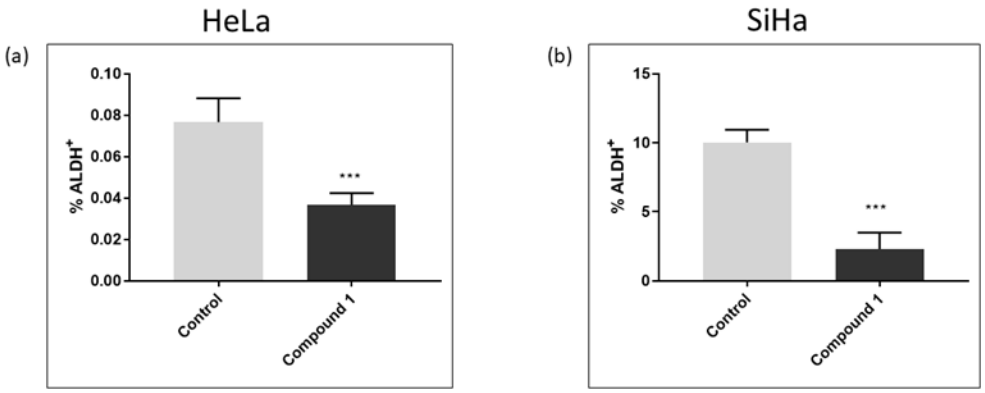

Figure S7. Cancer stem cells (ALDH+) after 48 h treatment with compound **1**. HeLa cell line (a) and SiHa cell line (b). Notes: Data represent are expressed as a mean from experiment performed in triplicate ± SD. Columns, mean of cells; bars, SD; p-values relate to a two-sample t-test comparing the treatment at the given concentration with when no treatment is applied (0 μM, control); *** p-value < 0.001.

Table S13. Calculated physiochemical properties of Compound 1 as determined by ADMET RankerTM.

**Property**	**Value**
cLogD	4.71
cLogP	6.91
pKa	10.07
pKb	3.62
Molecular Weight	539.01
Topological polar surface area	85.08
PAINS	Negative
Hepatotoxicity	Low Risk
AMES	Negative (high confidence)
hERG inhibition	Low Risk (high confidence)
Blood Brain Barrier diffusion	Positive (high confidence)
MDCK efflux	Low efflux
Hydrogen bond acceptors	5
Hydrogen bond donors	2
Number of rotatable bonds	5
Number of heteroatoms	8
Number of stereocentres	0

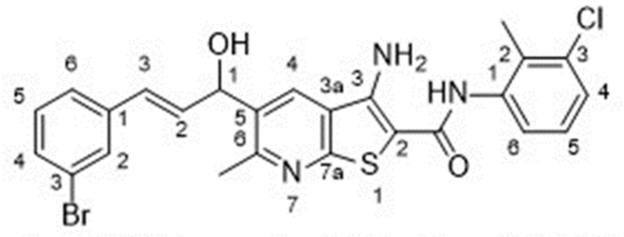

Figure S8. (E)-3-amino-5-(3-(3-bromophenyl)-1-hydroxyallyl)-*N*-(3-chloro-2-methylphenyl)-6-methylthieno[2,3-*b*]quinoline-2-carboxamide.

Table S14. The effect of (E)-3-amino-5-(3-(3-bromophenyl)-1-hydroxyallyl)-*N*-(3-chloro-2-methylphenyl)-6-methylthieno[2,3-*b*]quinoline-2-carboxamide on HeLa cell line

Cells were treated with 7 different concentrations of the for 4, 24, 48 and 72 h.

	p-value			
	4 h	24 h	48 h	72 h
0.05 μM	0.644	0.090	0.025	0.469
0.2 μM	0.280	0.699	0.009**	0.007
0.5 μM	0.878	0.917	0.039	0.046
1 μM	0.209	0.207	0.004**	0.417
2.5 μM	0.057	0.877	0.004**	0.158
5 μM	0.023	0.021	0.001**	<0.001***
10 μM	0.240	0.004	<0.001***	<0.001***

Note: p-values relate to a two-sample t-test comparing the treatment at the given concentration with when no treatment is applied (0 μM, control); ** p-value < 0.01; *** p-value < 0.001.

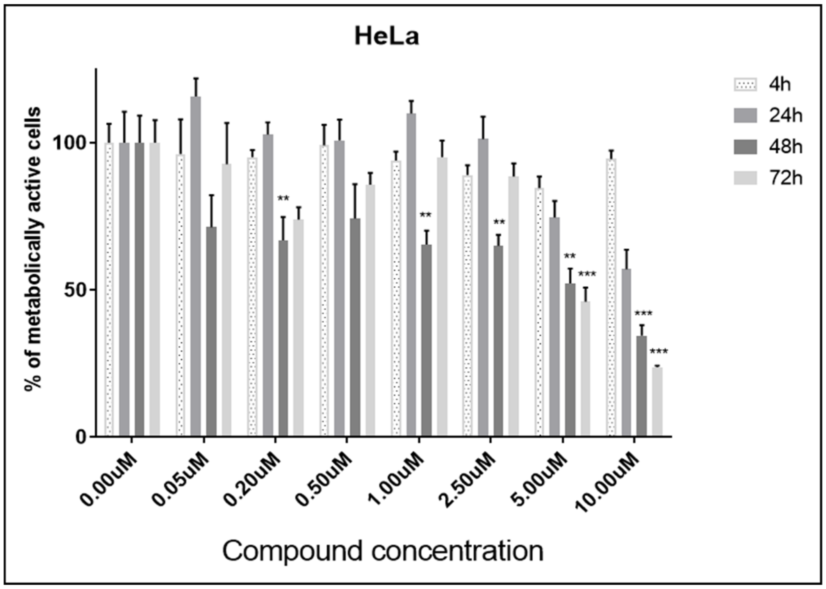

Figure S9. Cell viability of HeLa cell line after treatment with (E)-3-amino-5-(3-(3-bromophenyl)-1-hydroxyallyl)-*N*-(3-chloro-2-methylphenyl)-6-methylthieno[2,3-*b*]quinoline-2-carboxamide. *Notes:* Data are expressed as a mean from the experiment performed in triplicate ± SD. Columns, mean of metabolically active cells; bars, SD (standard deviation); * p-value < 0.05; ** p-value < 0.01; *** p-value < 0.001 (p-values relate to a two-sample t-test comparing the treatment at the given concentration with when no treatment is applied (0 μM).

Table S15. EC_{50} after 4, 24, 48 and 74 h after treatment with (E)-3-amino-5-(3-(3-bromophenyl)-1-hydroxyallyl)-*N*-(3-chloro-2-methylphenyl)-6-methylthieno[2,3-*b*]quinoline-2-carboxamide on HeLa cell line.

	4 h	24 h	48 h	72 h
EC_{50} / μM	ND	ND	3.621	5.105

Note: ND, could not be determined.

Table S16. The effect of (E)-3-amino-5-(3-(3-bromophenyl)-1-hydroxyallyl)-*N*-(3-chloro-2-methylphenyl)-6-methylthieno[2,3-*b*]quinoline-2-carboxamide on SiHa cell line

Cells were treated with 7 different concentrations for 24, 48 and 72 h.

	p-value		
	24 h	48 h	72 h
0.05 μM	0.159	0.991	0.149
0.2 μM	0.005**	<0.001***	<0.001***
0.5 μM	0.003**	<0.001***	<0.001***
1 μM	0.005**	<0.001***	<0.001***
2.5 μM	0.026*	0.001**	<0.001***
5 μM	0.002**	<0.001***	<0.001***
10 μM	0.003**	<0.001***	<0.001***

Note: p-values relate to a two-sample t-test comparing the treatment at the given concentration with when no treatment is applied (0 μM, control); * p-value < 0.05; ** p-value < 0.01; *** p-value < 0.001.

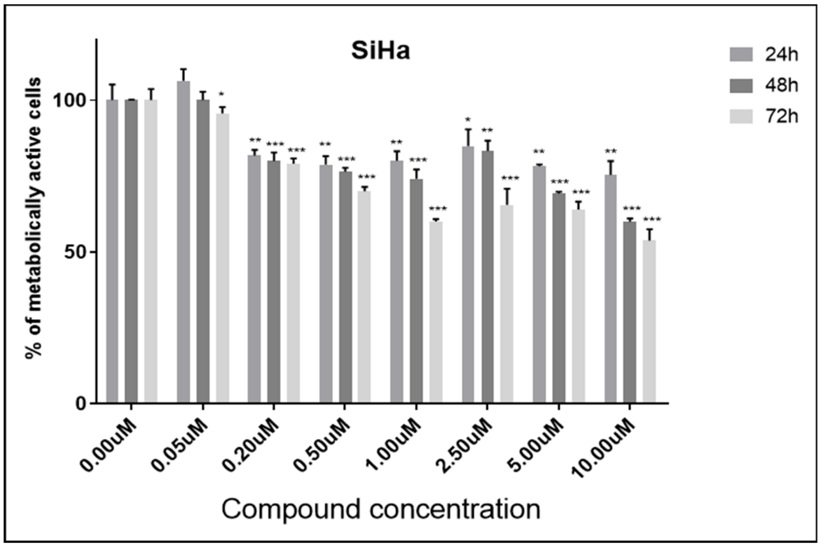

Figure S10. Cell viability of SiHa cell line after treatment with (E)-3-amino-5-(3-(3-bromophenyl)-1-hydroxyallyl)-*N*-(3-chloro-2-methylphenyl)-6-methylthieno[2,3-*b*]quinoline-2-carboxamide. *Notes:* Data are expressed as a mean from the experiment performed in triplicate ± SD. Columns, mean of metabolically active cells; bars, SD (standard deviation); * p-value < 0.05; ** p-value < 0.01; *** p-value < 0.001 (p-values relate to a two-sample t-test comparing the treatment at the given concentration with when no treatment is applied (0 μM).

Table S17. EC_{50} after 24, 48 and 74 h after treatment with (E)-3-amino-5-(3-(3-bromophenyl)-1-hydroxyallyl)-*N*-(3-chloro-2-methylphenyl)-6-methylthieno[2,3-*b*]quinoline-2-carboxamide on SiHa cell line.

	24 h	48 h	72 h
EC_{50} / μM	ND	ND	5.88

Note: ND, could not be determined.
